# Endophytic fungus *Pseudodidymocyrtis lobariellae* KL27 promotes taxol biosynthesis and accumulation in *Taxus chinensis*

**DOI:** 10.1186/s12870-021-03396-6

**Published:** 2022-01-03

**Authors:** Xiaoying Cao, Lingxia Xu, Jingyi Wang, Mengmeng Dong, Chunyan Xu, Guoyin Kai, Wen Wan, Jihong Jiang

**Affiliations:** 1 School of Life Science, Jiangsu Normal University, Xuzhou, Jiangsu 221116 People’s Republic of China; 2 Laboratory of Medicinal Plant Biotechnology, College of Pharmacy, Zhejiang Chinese Medical University, Hangzhou Zhejiang, 310053 People’s Republic of China

**Keywords:** *Taxus chinensis*, Taxol, Endophytic fungus, Fungus elicitor, Transcriptome

## Abstract

**Background:**

Taxol from *Taxus species* is a precious drug used for the treatment of cancer and can effectively inhibit the proliferation of cancer cells. However, the growth of *Taxus* plants is very slow and the content of taxol is quite low. Therefore, it is of great significance to improve the yield of taxol by modern biotechnology without destroying the wild forest resources. Endophytic fungus which symbiosis with their host plants can promote the growth and secondary metabolism of medicinal plants.

**Results:**

Here, an endophytic fungus KL27 was isolated from *T. chinensis*, and identified as *Pseudodidymocyrtis lobariellae*. The fermentation broth of KL27 (KL27-FB) could significantly promote the accumulation of taxol in needles of *T. chinensis*, reaching 0.361 ± 0.082 mg/g·DW (dry weight) at 7 days after KL27-FB treatment, which is 3.26-fold increase as compared to the control. The RNA-seq and qRT-PCR showed that KL27-FB could significantly increase the expression of key genes involved in the upstream pathway of terpene synthesis (such as *DXS* and *DXR*) and those in the taxol biosynthesis pathway (such as *GGPPS*, *TS, T5OH, TAT, T10OH, T14OH, T2OH, TBT, DBAT* and *PAM*), especially at the early stage of the stimulation. Moreover, the activation of jasmonic acid (JA) biosynthesis and JA signal transduction, and its crosstalk with other hormones, such as gibberellin acid (GA), ethylene (ET) and salicylic acid (SA), explained the elevation of most of the differential expressed genes related to taxol biosynthesis pathway. Moreover, TF (transcriptional factor)-encoding genes, including MYBs, ethylene-responsive transcription factors (ERFs) and basic/helix-loop-helix (bHLH), were detected as differential expressed genes after KL27-FB treatment, further suggested that the regulation of hormone signaling on genes of taxol biosynthesis was mediated by TFs.

**Conclusions:**

Our results indicated that fermentation broth of endophytic fungus KL27-FB could effectively enhance the accumulation of taxol in *T. chinensis* needles by regulating the phytohormone metabolism and signal transduction and further up-regulating the expression of multiple key genes involved in taxol biosynthesis. This study provides new insight into the regulatory mechanism of how endophytic fungus promotes the production and accumulation of taxol in *Taxus* sp.

**Supplementary Information:**

The online version contains supplementary material available at 10.1186/s12870-021-03396-6.

## Background

Taxol (generic name paclitaxel), a leading anticancer drug, is effectively used for the treatment of a wide range of cancers (eg, ovarian, breast, lung, Kaposiʼs sarcoma, cervical, and pancreatic cancers) [1, 2]. Taxol was originally isolated from the bark of the Pacific yew (*Taxus brevifolia*). And now can be mainly derived from plants in genus *Taxus*. *Taxus* sp. is a tall evergreen tree or shrub of *Taxaceae*. It is a valuable timber tree specie, and remarkable with its horticultural and ornamental value, and medicinal value. However, due to the increasing incidence of cancer and very slow growth and low productivity of *Taxus* sp., the production of taxol is not sufficient to meet increasing market demands [3]. Therefore, it is extremely urgent to find cost-effective alternatives sources or methods, that can increase the production and accumulation of taxol in *Taxus* trees without harm for the human environment. Several strategies, including chemical synthesis of taxol [4], plant cell culture, metabolic engineering [5] and taxol-producing microbes [6] were adopted to improve taxol productivity*.* However, due to a variety of adverse factors, these strategies didn’t provide an effective instrument to solve the growing imbalance between supply and demand of taxol yet. Currently, the needles of yew plants is one of the two main sources for taxol and its precursor, and the other sources is *Taxus* suspension cell cultures [7–11]. So, looking for methods to improve the taxol yield in needles of *Taxus* trees is also a feasible way to solve the imbalance in the supply and demand of taxol.

There are various endophytes in medicinal plants, and those endophytes are mainly exist in the intercellular space of plant tissues. Endophyte and its host plants formed a harmonious symbiotic relationship during the long-term co-evolution process. Especially, increasing evidences showed that endophyte can directly and indirectly promote the growth and secondary metabolites of its host plants through various ways [12–14]. For example, endophyte can induced the development of its host plants by directly producing plant growth hormone themselves [12], or indirectly by promoting its host plants capacity of nutrients absorption and stress resistance [13]. Furthermore, endophyte can also produce bioactive compounds which are the same or similar to the secondary metabolites in its hosts [14]. Since the firstly reported taxol-producing endophytic fungus *Taxomyce andreanae* isolated from the bark of *T. brevifolia* in 1993 [6], about 200 endophytic fungus belonging to more than 40 fungal genera had been reported to produce taxol till now [11, 15]. Zhou et al. [16]. identified three taxol-producing endophytic fungi from 38 endophytic fungal strains isolated from *T. chinensis* var. *mairei* by the aseptic technique. Gangadevi and Muthumary [17] isolated a taxol-producing endophytic fungus *Bartaliniarobil lardoides* (strain AMB-9) from a medicinal plant *Aegle marmelos*. The yield of taxol of this stain reach to 187.6 μg/L. Recently, El-Sayed et al. [18] immobilized *Aspergillus fumigatus* TXD105-GM6 and *Alternaria tenuissima* TER995-GM3 in calcium alginate beads for the production of taxol in shake flask cultures, reaching to 4540.14 μg/L by TXD105-GM6 and 2450.27 μg/L by TER995-GM3, which is the highest report by academic laboratories for microbial cultures using endophytic fungus for taxol production. Furthermore, endophyte can also produce special chemicals as endophyte elicitors, which induce and stimulate the secondary metabolism of their hosts [19]. Hemmati et al. [20] screened endophytes from *Catharanthus roseus*, and found that some endophytes could induce biosynthesis and accumulation of ajmalicine and vinblastine in the host plants. Wang et al. [21] used endophytic fungus of *Artemisia annua* to prepare elicitors, which promoted the biosynthesis of artemisinin in host plants. Compared with the control, the yield of artemisinin increased by more than 50%. Wang et al. [22] isolated an endophytic fungus, *Aspergillus niger*, from the inner bark of *T. chinensis* tree, could stimulate the taxol accumulation in *T. chinensis* cell suspension culture.

RNA-seq, a cost-effective and highly accurate DNA sequencing technology, has been frequently used to evaluate the functional complexity of transcriptomes after treatments of various situations [23]. Currently, RNA-seq has also been widely applied to investigating the taxol biosynthesis in different *Taxus* species, including tissue-specific transcriptomes [24], interspecific transcriptomics [25] and transcriptional profile response of elicitation with methyl jasmonic acid (MeJA) [26]. Although, many studies pointed out that endophytic fungus can promote the growth and secondary metabolism in *T. chinensis*, but most of them were focused on the diversity and promoting ability of endophytic fungus on the growth of *T. chinensis.* There are only a few studies on investigation of endophytic fungus effect of taxol accumulation and its action mechanisms. In early study, we isolated an endophytic fungus *P. lobariellae* KL27 from *T. chinensis*, which can promote the taxol accumulation in the needles of *T. chinensis*. In this study, our objective was to decipher the mechanism of influences on the taxol biosynthesis and accumulation caused by the endophytic fungus *P. lobariellae* in *T. chinensis* needles by RNA-seq technology. So as to provide a theoretical basis for the study of endophytic fungus regulating the accumulation of medicinal components of *T. chinensis* and to lay the foundation for its further practical utilization.

## Methods

### Preparation of fermentation broth of KL27 and treated of *T. chinensis* needles

KL27 was incubated on PDA slant medium and incubated at 28°C for 7 days, then transferred to PDB liquid medium and incubated at the shaking speed of 180 rpm at 28°C for 7 d. Then, the fermentation broth of KL27 (KL27-FB) was collected. After sterilization of KL27-FB and PDB (set as control) by filtrating through 0.45 μm sterilized filters, they were spread evenly on the surface of needles of five-year old *T. chinensis* respectively in a growth chamber of Jiangsu Normal University, Xuzhou, China*.* The growth conditions were set at 25 °C with a light/dark cycle of 16/8 h and a 50 ~ 60% relative humidity. Seedlings of each treatment were separately into two parts. At 0.5 h and 6 h after the KL27-FB treatments, one part of the seedings is harvested and frozen in liquid nitrogen and sent for RNA sequencing. Then, the other part of seedlings was harvested for taxanes analysis at 7 d after KL27-FB treatments. Each treatment was performed with three biological replicates.

### HPLC analysis of taxanes

Taxanes were extracted and detected referred to the literature [27] with minor modifications. In briefly, needles of *T. chinensis* from every treatment were freeze-dried and powdered. Then, the powder was passed through a filter (0.42 mm pore size). 1.0 g filtered powder was mixed with 30 ml of 100% methanol and then ultrasonicated for 60 min and 3 times. After centrifugation at 5000 rpm for 5 min, the supernatant liquor was collected and extracted with dichloromethane/water (1:1, v/v) for 3 times. The organic fraction was collected, dried under vacuum and resuspended in 1 ml methanol and filtered through a 0.45 μm organic phase filter. 10-deacetylbaccatin III, baccatin III and taxol content in the methanol sample solution were analyzed by HPLC using a C18 column (Hypersil ODS2 4.6 × 200 mm, 5 μm) with detection at 227 nm. Column temperature was 25 °C. The mobile phase was a mixture of 0.1% formic acid solution and acetonitrile, and flow rate was at 1 ml/min. Acetonitrile (solvent A) and 0.1% formic acid solution (solvent B) as mobile phase with a linear gradient was used: 0 min: 5% A; 25 min: 100% A; 30 min: 100% A; 35 min: 5% A. The quantification of 10-deacetylbaccatin III, baccatin III and taxol were based on external standards (Sigma, St. Louis, USA).

### RNA extraction

Total RNA was extracted using the mirVana miRNA Isolation Kit (Invitrogen, USA) following the manufacturer’s protocol. RNA purity and quantification were evaluated using 1% agarose gel electrophoresis and the NanoDrop 2000 spectrophotometer (Agilent Technologies, Santa Clara, CA). RNA integrity was assessed using the Agilent 2100 Bioanalyzer (Agilent Technologies, Santa Clara, CA).

### Library construction and sequencing

Total RNA samples of 10 μg of each RNA extract (4 treatments × 3 biological replicates) were prepared. Then libraries were constructed using TruSeq Stranded mRNA LT Sample Prep Kit (Illumina, San Diego, CA, USA) according to its manual. The transcriptome sequencing were conducted by OE Biotech Co., Ltd. (Shanghai, China). Sequencing was carried out using Illumina HiSeq X Ten platform according to its instruction.

### De novo assembly and read annotation

Raw data (raw reads) of fastq format was firstly processed using Trimmomatic [28]. The reads containing ploy-N and the low quality reads with low Q-value (≤30) bases were removed by the Perl program (version 5.18.4). Clean reads of twelve RNA samples were merged and de novo assembled using Trinity Package 2.4 with paired-end method [29].

### Functional annotation and enrichment analysis

The unigenes were annotated by alignment of the unigenes with the NCBI nonredundant (Nr) database (https://www.ncbi.nlm.nih.gov), the Swiss-Prot database (https://www.expasy.ch/sprot), the evolutionary genealogy of genes: Non-supervised Orthologous Groups (eggNOG) and Clusters of orthologous groups for eukaryotic complete genomes (KOG) database (https://www.ncbi.nlm.nih.gov/COG) using Diamond [30] with a threshold e-value of 10^− 5^. The proteins with the highest hits to the unigenes were used to assign functional annotations. The unigenes were also mapped to the Kyoto Encyclopedia of Genes and Genomes (KEGG) database (https://www.genome.jp/kegg) and Gene Ontology (GO) classifications by Blast2GO (https://www.blast2go.com/) to annotate the potential metabolic pathways. Hierarchical cluster analysis of differential expressed unigenes (DEGs) was performed to demonstrate the expression pattern of genes in different groups and samples. GO enrichment and KEGG pathway enrichment analysis of DEGs were performed respectively using R based on the hypergeometric distribution.

### Differentially expressed unigene analysis

After annotation, Fragments per kilobase per million (FPKM) and read counts value of each unigene were calculated using Bowtie2 [31] and eXpress [32]. DEGs between different groups were identified using the DESeq functions estimate size factors and nbinom test. *p* value < 0.05 and foldChange > 2 or foldChange < 0.5 were set as the threshold for significantly differential expression. False discovery rate (FDR) was used as the threshold of *p*-value in multiple test to judge the significance of gene expression difference.

### Quantitative real-time PCR (qRT-PCR) validation

The needles of *T. chinensis* were collected at 0.5 and 6 h after treated with KL27-FB and PDB as control. Total RNA of the needles samples of *T. chinensis* were extracted using an RNApure plant kit (Aidlab, Beijing, China) according to the instructions. The first strand of cDNA was synthesized with 1 μg RNA using the HiScript® Q RT SuperMix for qPCR (+ gDNA wiper) (Vazyme, China) according to its manuals. RT-qPCR was carried out with ChamQ SYBR qPCR Master Mix (Vazyme, China) following the manufacturer’s instructions. In addition, primers were designed according to the software of Primer 6. The sequences were listed in additional file 1. To analyze the expression levels of genes using qRT-PCR, each reaction was carried out in a total volume of 20 μl, which contained 2 μl of cDNA. The PCR program was set as follow: initial denaturation of 95 °C for 30 s, 40 cycles of denaturation at 95 °C for 10 s and annealing and extension at 60 °C for 30 s, and a melting curve was obtained at 95 °C for 15 s and at 60 °C for 1 min followed by continuous heating on the StepOne Plus Real-Time PCR System (Applied Biosystems, USA). After qRT-PCR, melting curves were generated to test specificity of the products. Data were derived from three independent biological replicates. With *T. chinensis GAPDH* gene as the internal reference gene, 2 ^−△△CT^ method was used to analyze the relative gene expression level.

## Results

### KL27-FB promote accumulation of taxol in *T.chinensis* needles

**Fig. 1:**
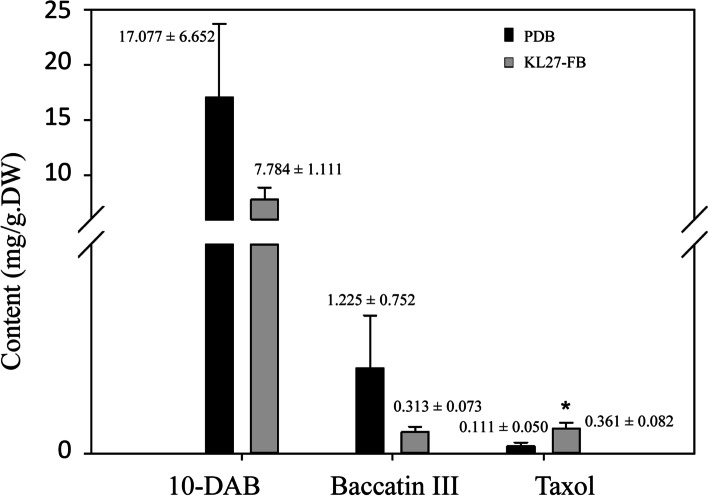
Effects of KL27-FB on the contents of taxanes in *T.chinensis* needles. Significant variation (0.01 < *p <* 0.05) is indicated by *. Error bars represent means ± SD (*n* = 3)

To investigate the potential effect of KL27-FB on the taxol biosynthesis in needles of *T.chinensis* seedlings, the needles taxanes contents with or without KL27-FB treatment were determined. The results showed that KL27-FB could significantly increase the accumulation of taxol in *T.chinensis* needles (Fig. 1). After treated with KL27-FB, the taxol content increased by 326% of the control group (*p* < 0.05), while the the contents of 10-deacetylbaccatin III and baccatin III decreased by 54.42 and 74.43%, respectively. These results indicated that KL27-FB could significantly induce the conversion of precursors to end product taxol in taxol biosynthesis of *T.chinensis* needles. And the taxol content ever reached to 0.361 ± 0.082 mg/g·DW.

### Illumina sequencing, sequence assembly and read annotation

As shown in Fig. 1, KL27-FB treatment caused a substantial change in the abundance of taxol, 10-deacetylbaccatin III and baccatin III. To gain a comprehensive overview of responsive genes, we carried out a transcriptomic sequencing of needles of five-year old *T.chinensis* seedling after KL27-FB treatment at 0.5 h and 6 h, respectively. Three biological repeats were prepared for each condition. Using the next-generation sequencing platform (Illumina), RNA-seq datas from the controls at 0.5 h after PDB treatment (CK0.5H), samples at 0.5 h after K27-FB treatment (Y0.5H), the controls at 6 h after PDB treatment (CK6H) and samples at 6 h after K27-FB treatment (Y6H) were collected.

**Fig. 2:**
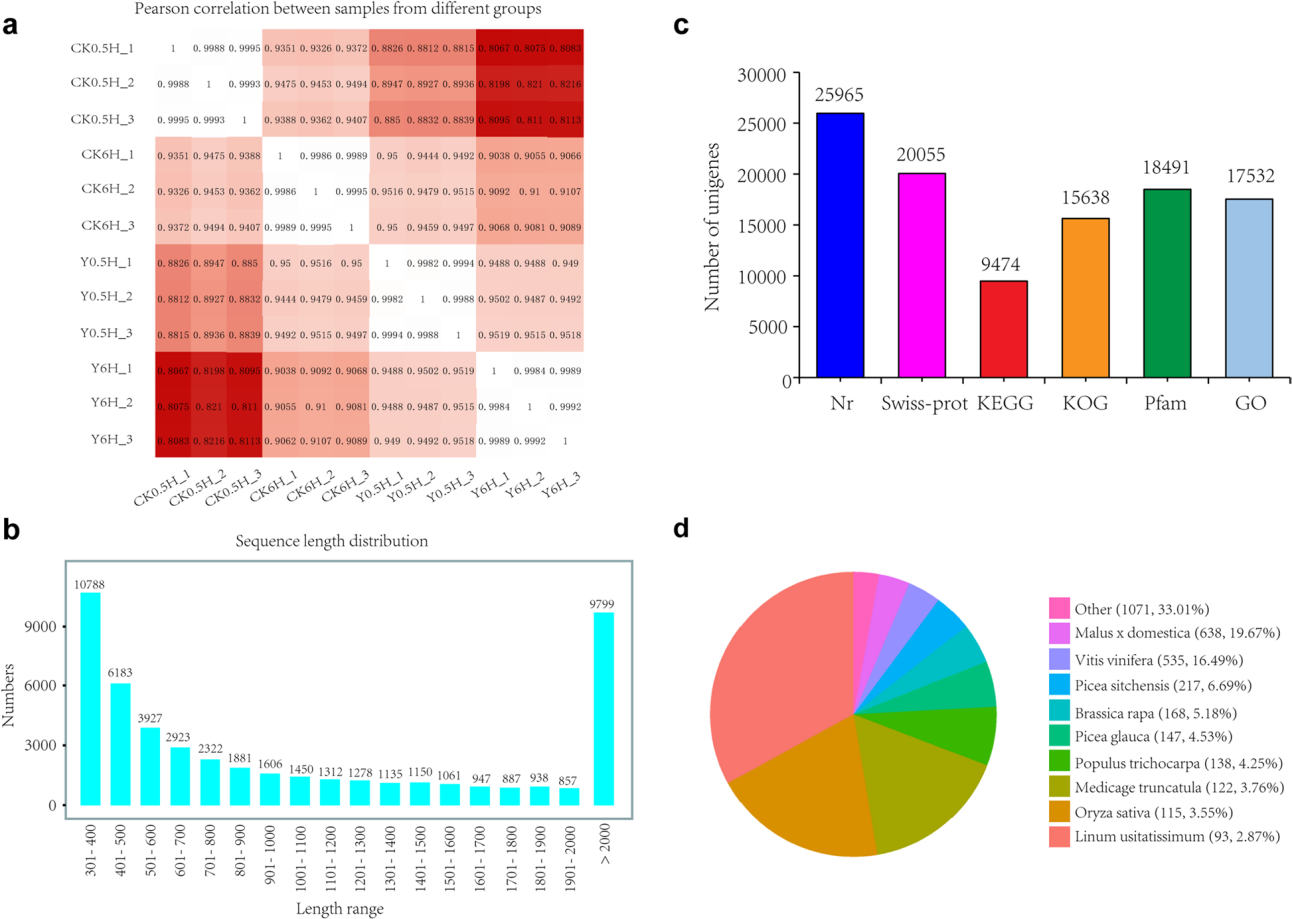
Illumina sequencing and transcriptomes of *T.chinensis* needles with various treatments. **a** Pair-wise pearson’s correlation coefficients of the sequencing data from four groups x three replicates. **b** The size distributions of unigenes of *T.chinensis* needles. **c** The annotation of unigenes basing on various databases. **d** The species distribution of the annotated unigenes

The raw reads were qualified trimmed (threshold Q30), and adapters were removed, yielding 83.61 Gb of sequence date, including 22.81 Gb from CK0.5H, 19.23 Gb from Y0.5H, 19.97 Gb from CK6H and 21.61 Gb from Y6H. Among raw reads in all samples, the Q30 values ranged from 93.05 to 93.75%, and the GC content ranged from 45.10 to 45.87% (Additional file 2). As shown in Fig. 2a, pair-wise pearson’ s correlation coefficients of three replicates x four groups showed high repeatability of the sequencing data. A principal components analysis (PCA) was performed to analysis the transcriptomic variations, and the explained values of PC1 and PC2 were 73.26 and 23.48%, respectively (Additional file 3). The PCA clearly separated the four samples into three groups, the close similarity between CK0.5H and CK6H suggested PDB treatment had only a minor effect on the transcriptomes of *T.chinensis*. However, KL27-FB could significantly effect the transcriptomes of *T.chinensis* and the transcriptomes were significantly changed after KL27-FB treatment over time.

Thereafter, all clean reads from the four groups were assembled into 50,444 unigenes (Additional file 4) with a mean length of 1239 bp and N50 size of 1992 bp. The size distributions of unigenes were shown in Fig. 2b, 19.438% of the reads were > 2000 bp in length, and the majority of the reads (57.74%) were < 1000 bp in length. Gene annotation was performed to predict the functions of the unigenes. The unigenes were searched against the Nr, SwissProt, KEGG, KOG, Pfam and GO databases, and there were 25,956 unigenes (51.46%) matching the protein sequences in the Nr database, 20,055 (39.76%) in the SwissProt database, 9474 (18.78%) in the KEGG database, 15,638 (31.00%) in the KOG database, 18,491 (36.66%) in the Pfam database and 17,532 (34.76%) in the GO database (Fig. 2c). The species distribution of the annotated unigenes was shown in Fig. 2d. A number of unigenes in *T. chinensis* showed high similarity to genes in the other species. The largest number of *Taxus* homologous genes were identified in *Malus* X *domestica*.

GO and KEGG terms of the sequencing data were analyzed to classify the functions of predicted unigenes. For GO analysis, there were 17,532 unigenes that were annotated with Bowtile2 and were categorized into 53 functional groups in the three categories of biological process, cellular component, and molecular function. Among them, the seven most presented GO groups were “cell”, “cell part”, “cellular process”, “metabolic process”, “organelle”, “binding” and “catalytic activity” (Additional file 5). For KEGG analysis, the 9474 unigenes were categorized into 20 functional groups in the six categories of cellular processes, environmental information processing, genetic information processing, human diseases, metabolism and organismal systems. Among them, the most five presented KEGG groups were “Translation”, “Carbohydrate metabolism”, “Folding, sorting and degradation”, “Enzyme metabolism” and “Amino acid metabolism” (Additional file 5). Then KOG database were used to evaluate the integrality of the transcriptome library. In total, 15,638 out of 50,444 unigenes were divided into 25 different KOG categories and the three most represented largest groups were R, O and J category which presented “general function prediction only”, “posttranslational modification, protein turnover, chaperones” and “Translation, ribosomal structure and biogenesis” respectively (Additional file 5).

### GO and KEGG enrichment analysis of DEGs

**Fig. 3:**
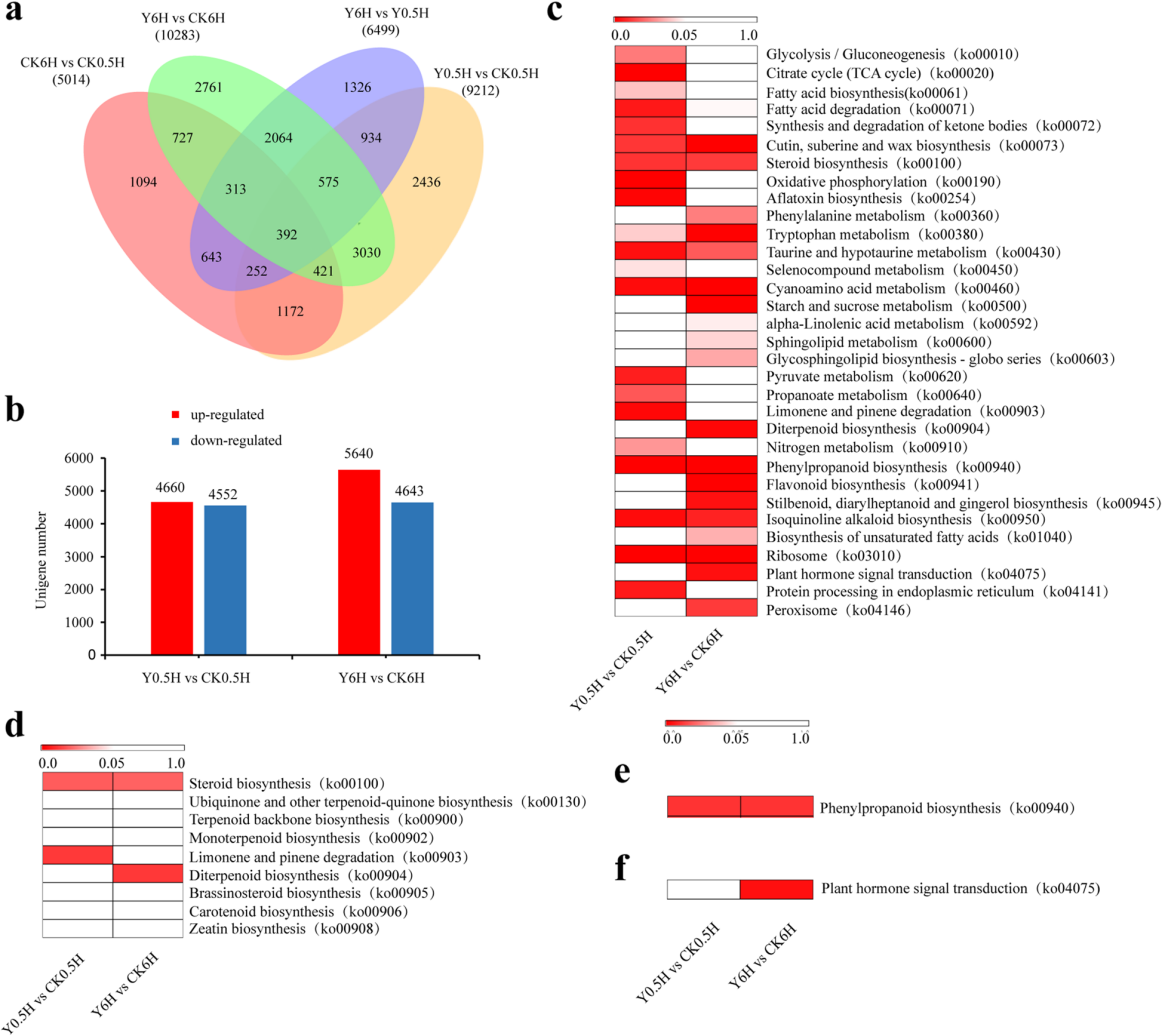
Identification of the DEGs among *T.chinensis* needles at 0.5 h and 6 h after KL27-FB treatment. **a** A venn diagram showed the number of genes in four comparisons, including CK6H vs CK0.5H, Y0.5H vs CK0.5H, Y6H vs CK6H and Y6H vs Y0.5H comparisons. **b** The numbers of up- and down-regulated unigenes in the two comparisons. KEGG enrichment analysis of the DEGs in the two comparisons. KEGG enrichment analysis of the DEGs related to terpenoid biosynthesis (**d**), phenylpropanoid biosynthesis (**e**) and plant hormone signal transduction (**f**). The significant *p* value of each KEGG term in the two comparisons were shown by heatmaps. The bar indicated the significant values

In this study, the numbers of DEGs identified in each groups were shown in a venn diagram (Fig. 3a). In detail, 4660 up-expressed unigenes and 4552 down-expressed unigenes were identified in the Y0.5H vs CK0.5H comparison, and 5640 up-expressed unigenes and 4643 down-expressed unigenes were identified in the Y6H vs CK6H comparison (Fig. 3b). GO and KEGG classifications were performed for a preliminary insights into the proteomic differences in *T. chinensis* needle cells after KL27-FB treatment. A total of 17,532 prominently expressed unigenes assigned to 7202 GO terms were identified from the *T.chinensis* needles RNA-seq data. After KL27-FB treatments, most of the DEGs were significantly enriched in seven GO categories. The most highly represented terms in the biological processes, cellular component, and molecular function category were “cellular process” and “metabolism process”, “cell” “cell part” and “organelle”, and “binding” and “catalytic activity”, respectively (Additional files 6 and 7). Among them, 1172 and 953 GO terms were significantly enriched (*p* < 0.05) at 0.5 h and 6 h respectively after KL27-FB treatment (Additional files 6 and 7). In total, 9474 prominently expressed unigenes assigned to 126 KEGG canonical pathways were identified from the *T.chinensis* needles in our RNA-seq data. Among them, the three most represented pathways were “Ribosome”, “Protein processing in endoplasmic reticulum” and “Oxidative phosphorylation”. Moreover, among these DEGs associated KEGG pathways, 21 and 20 pathways were significantly enriched (p < 0.05) at 0.5 h and 6 h respectively after KL27-FB treatment (Fig. 3c).

### KL27-FB increased terpenoid biosynthesis

Terpenoids, which consists the most abundant and structurally diverse group of plant secondary metabolism, are playing important roles in protect plants against pathogenic attacks and defense response to environmental stresses [33]. And in plants, all terpenoids are derived from the basic isoprene, such as isopentenyl diphosphate (IPP) and dimethylallyl diphosphate (DMAPP) [34]. There are nine terpenoid biosynthesis-related KEGG pathways, including “steroid biosynthesis” (ko00100), “ubiquinone and other terpenoid-quinone biosynthesis” (ko00130), “terpenoid backbone biosynthesis” (ko00900), “monoterpenoid biosynthesis” (ko00902), “limonene and pinene degradation” (ko00903), “diterpenoid biosynthesis” (ko00904), “brassinosteroid biosynthesis” (ko00905), “carotenoid biosynthesis” (ko00906) and “zeatin biosynthesis” (ko00908), helpful to analysis the differential expression of terpenoid biosynthesis-related genes after KL27-FB treatment. In detail, the genes in 2 KEGG pathways, including ko00100 (*p* = 0.0101) and ko00903 (*p* = 0.00156), were significantly enriched at 0.5 h after KL27-FB treatment (Fig. 3d). And genes in 2 KEGG pathways, including ko00100 (*p* = 0.011) and ko00904 (*p* = 0.0012), were significantly enriched at 6 h after KL27-FB treatment (Fig. 3d). Additionally, the RNA-seq data revealed that 208 genes were annotated as terpenoid biosynthesis pathway members. Among them, 49 unigenes, including 19 and 17 DEGs, were involved in the steroid biosynthesis; 64 unigenes, including 10 and 12 DEGs, were involved in the terpenoid backbone biosynthesis, 15 unigenes, including 5 and 4 DEGs, were involved in the monoterpenoid biosynthesis, 38 unigenes, including 10 and 16 DEGs, were involved in the diterpenoid biosynthesis, 32 unigenes, including 3 and 6 DEGs, were involved in the carotenoid biosynthesis, at 0.5 h and 6 h after KL27-FB treatment, respectively (Additional file 8). These results indicated that abundant of genes involved in the terpenoids biosynthesis were effected by the KL27-FB stimuli.

**Fig. 4:**
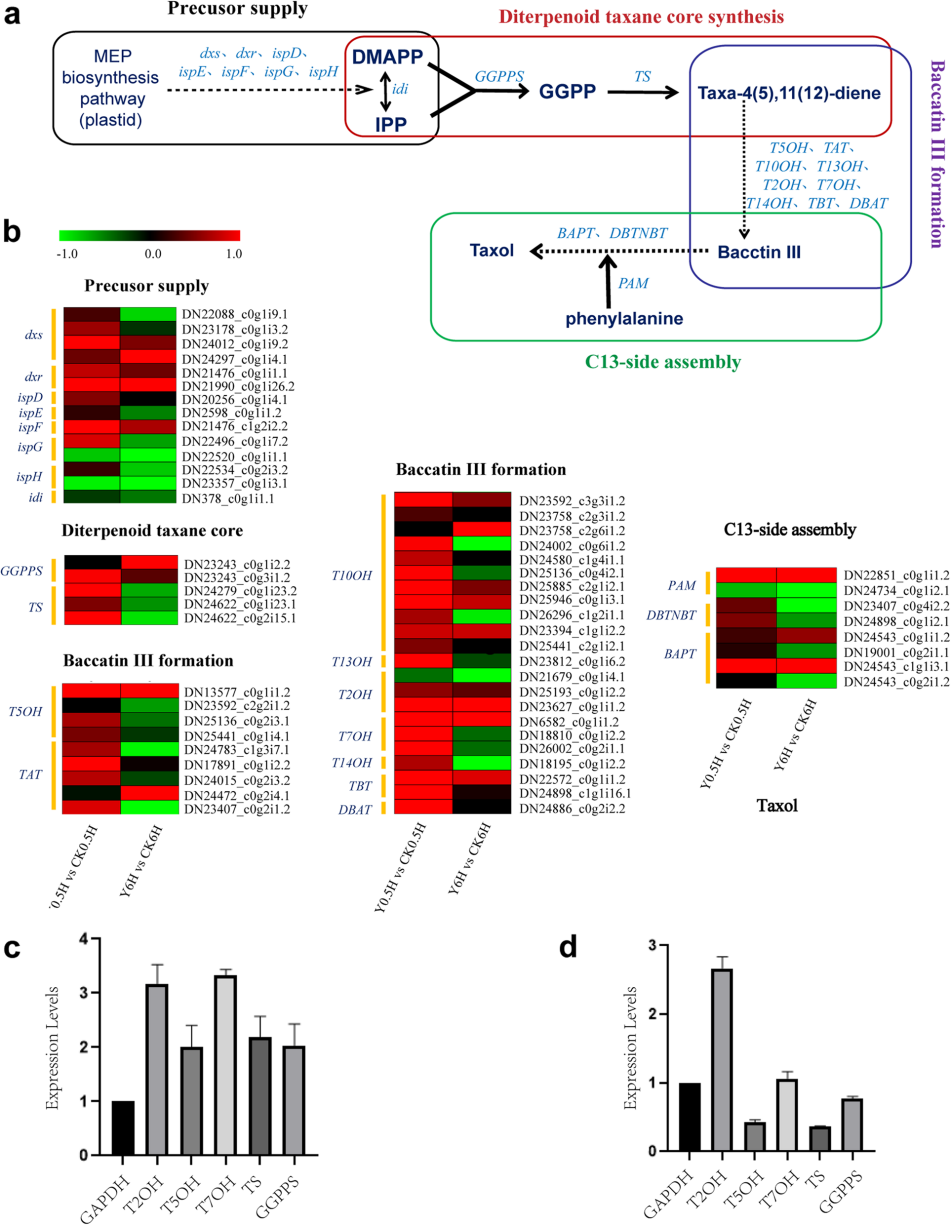
Differential expression of the taxol biosynthesis-related unigenes. **a** Overview of the taxol biosynthesis pathway. **b** Expression analysis of the taxol biosyntheisis-related unigenes. qRT-PCR Validation of six DEGs in taxol biosynthesis pathways at 0.5 h (**c**) and 6 h (**d**) after KL27-FB treatments. Enzymes abbreviations are: dxs: 1-deoxy-D-xylulose5-phosphate synthase; dxr: 1-deoxy-xylulose5-phosphate reductoisomerase; ispD: 2-C-methyl-D-erythritol 4-phosphate cytidylyltransferase; ispE: 4-diphosphocytidyl-2-C-methyl-D-erythritol kinase; ispF: 2-C-methyl-D-erythritol 2,4-cyclodiphosphate synthase; ispG: (E)-4-hydroxy-3-methylbut-2-enyl-diphosphate synthase; ispH: 4-hydroxy-3-methylbut-2-en-1-yl diphosphate reductase; idi: isopentenyl-diphosphate Delta-isomerase; GGPPS: geranylgeranyl diphosphate synthase; TS: taxadiene synthase; T5OH: taxane 5a-hydroxylase; TAT: taxadiene-5α-ol-O-acetyl transferase; T10OH: taxane 10 β-hydroxylase; T13OH: taxane 13 α-hydroxylase; T2OH: taxane 2 α-hydroxylase; T7OH: taxane 7 β-hydroxylase; T14OH: taxane 14 β-hydroxylase; TBT: taxane-2α-O-benzoyltransferase; DBAT: 10-deacetyl baccatin III-10-O-acetyltransferase; PAM: phenylalanine aminomutase; BAPT: 3-amino-3-phenylpropanoyltransferase; DBTNBT: 3′-N-debenzoyltaxol N-benzoyltransferase. The bar indicated the “log2(fold change)”

In *Taxus* sp., the precursor of the diterpenoid taxane core, geranylgeranyl diphosphate (GGPP), is synthesized from the C5 isoprenoid precursor IPP and DMAPP, which are produced by the plastid-localized plastidial 2-C-methyl-D-erythritol 4-phosphate (MEP) pathway [34]. So analysis the change of genes involved in terpenoid biosynthesis and taxol biosynthesis after KL27-FB treatment is helpful to investigate the molecular mechanism of taxol accumulation responding to KL27-FB stimuli in *T. chinensis* needles. Genes involved in the biosynthesis of IPP and DMAPP by MEP pathway were mapped in the RNA-seq data of *T. chinensis* needles, and several unigenes corresponding to these genes were presented and showed up-regulated after KL27-FB stimuli (Fig. 4b). Especially, two genes encoding the two enzymes catalyze the slow steps of the MEP pathway, *DXS* and *DXR* were significantly up-regulated after KL27-FB treatment (Fig. 4b), indicated that KL27-FB elicitor could improve the precursor supply for diterpenoid taxane core synthesis in taxol biosynthesis pathway.

### KL27-FB effected phenylpropanoid biosynthesis

Phenylpropane biosynthesis is one of the most important secondary metabolic pathways in plants, producing more than 8000 metabolites, which plays an important role in plant growth and development and plant-environmental interactions [35]. In this study, based on KEGG analysis the significant values of KEGG pathway “phenylpropanoid biosynthesis” (ko00940) were 8.79E-05 and 1.05E-12 at 0.5 h and 6 h after KL27-FB treatments respectively, which showed that phenylpropanoid biosynthesis was significantly activated after KL27-FB elicitation (Fig. 3e). Our RNA-seq data also shown that 165 unigenes, including 62 and 81 DEGs at 0.5 h and 6 h after KL27-FB elicitation respectively, were annotated as phenylpropanoid biosynthesis members (Additional file 8). Among these unigenes, the expressions of 37 DEGs were up-regulated, and 25 DEGs were down-regulated at 0.5 h after KL27-FB treatment. While, the expressions of 42 DEGs were up-regulated, and 39 DEGs were down-regulated at 6 h after KL27-FB elicitor (Additional file 9). Genes related to key enzymes in the phenylpropanoids biosynthesis pathways [35], including phenylalanine ammonia-lyase (PAL), PAM, 4-coumarate CoA ligase (4CL), trans-cinnamate 4-monooxygenase, caffeic acid 3-O-methyltransferase (COMT), shikimate O-hydroxy cinnamoyltransferase (HCT), *p*-coumarate 3-hydroxylase (C3’H) *et. al* were differently expressed in *T. chinensis* needles after KL27-FB treatments (Additional file 9). These results suggested that KL27-FB significantly affected the phenylpropanoid biosynthesis in *T. chinensis* needles.

Additionally, The phenylpropanoid biosynthesis pathway provides the C13-phenylpropanoid side chain for taxol biosynthesis. To provide insight into the effects of KL2-FB on the genes involved in both phenylpropanoid biosynthesis and taxol biosynthesis in *T. chinensis* needles. The expression pattern of *PAM* gene after KL27-FB treatment over time was analyzed. As shown in Fig. 4b, the expression of a unigene (DN22851_c0g1i1.2) corresponding to *PAM* were highly regulated after KL27-FB treatment, which suggested that the KL2-FB may improve the C13-phenylpropanoid side chain supply and increase the accumulation of taxol in *T. chinensis* needles.

### KL27-FB activated the taxol biosynthesis pathway

**Fig. 5:**
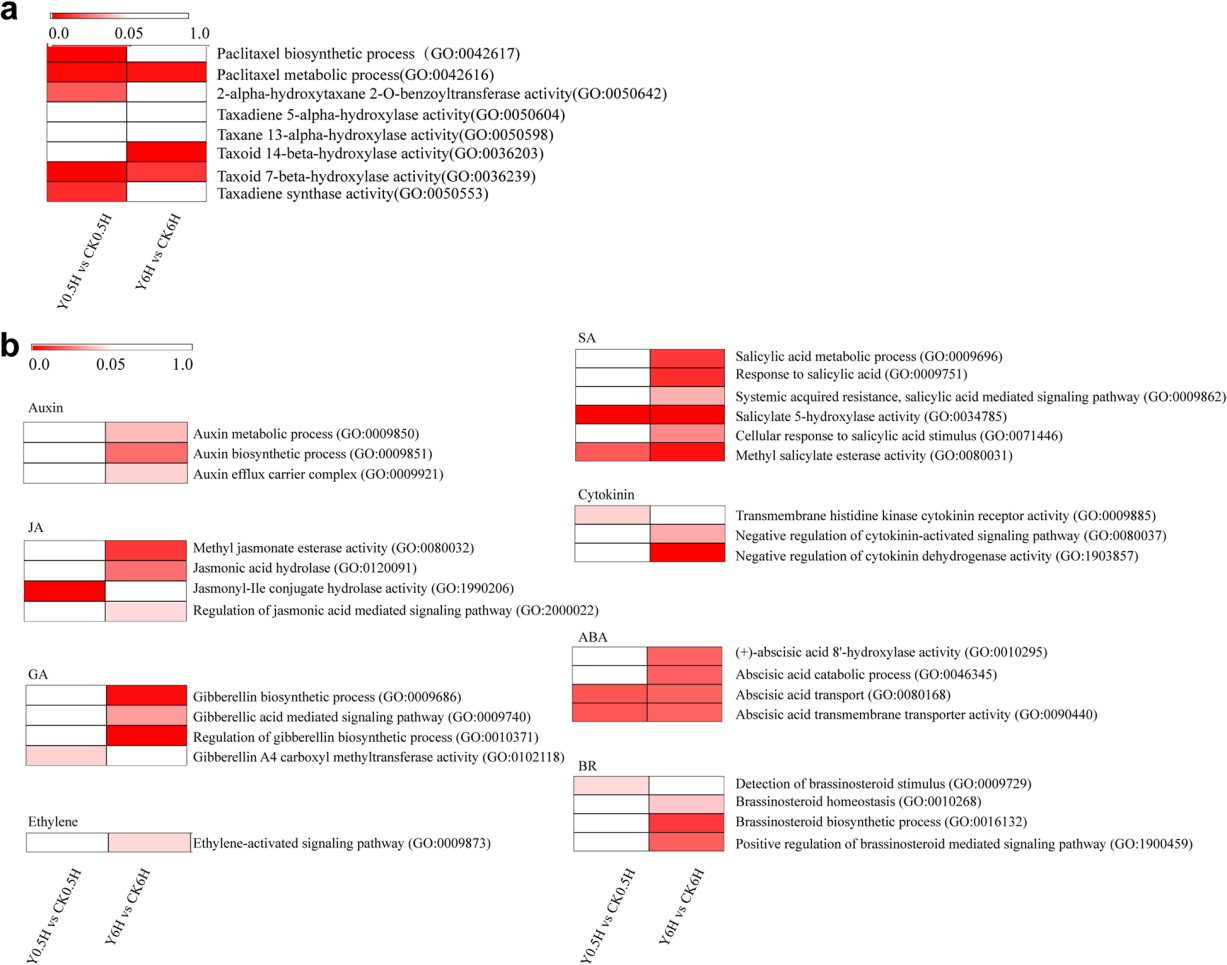
GO enrichment of terms related to taxol biosynthesis (**a**) and hormone signal (**b**). The bar indicated the “*p* value”

In this study, there were eight taxol biosynthesis-related GO terms, including “paclitaxel biosynthetic process” (GO:0042617), “paclitaxel metabolic process” (GO:0042616), “2-alpha-hydroxytaxane 2-O-benzoyltransferase activity” (GO:0050642), “taxadiene 5-alpha-hydroxylase activity” (GO:0050604), “taxane 13-alpha-hydroxylase activity” (GO:0050598), “taxoid 14-beta-hydroxylase activity” (GO:0036203), “taxoid 7beta-hydroxylase activity” (GO:0036239) and “taxadiene synthase activity” (GO:0050553), presented in our transcriptome data, which is helpful to analysis the differential expression of taxol biosynthesis -related genes after KL27-FB treatment. In detail, the genes in 5 GO terms, including GO:0042617 (*P* = 1.14E^− 05^), GO:0042616 (*P* = 0.0017), GO:0050642 (*P* = 0.0177), GO:0036239 (*P* = 0.0003) and GO:0050553 (*P* = 0.0083), were significantly enriched in the Y0.5H vs CK0.5H comparison (Fig. 5a). While, the genes in 3 GO terms, including GO:0042616 (*P* = 0.0029), GO:0036203 (*P* = 0.0000), and GO:0036239 (*P* = 0.0109) were significantly enriched in the Y6H vs CK6H comparison (Fig. 5a). These results suggested that *T. chinensis* needle cells could rapidly response to the KL27-FB stimuli and adjusted the taxol biosynthesis.

At presently, the taxol biosynthesis pathway has been basically revealed [34]. In *Taxus* sp., the pathway toward taxol involves about 19 steps of enzymatic reaction, generally divide into three main stages. The first stage, the diterpenoid taxane core synthesis, which mainly concerns the cyclization of GGPP to taxa-4 [5],11[12]-diene conducted by taxadiene synthase (TS) [36, 37]. Secondly, baccatin III formation, in this course, taxadiene goes through a series of enzymatic reaction including acylation, hydroxylation and transferase to form baccatin III [38]. Lastly, C13-side chain assembly, the C13-side chain is synthesized and attached to baccatin III to form taxol [39]. In briefly, for taxol biosynthesis, four intermediate steps, including precursor supplement, diterpenoid taxane core synthesis, baccatin III formation and C13-side chain assembly, are involved (Fig. 4a).

To further analyze how these DEGs contribute to the higher taxol accumulation after KL27-FB treatment, the expression patterns of genes involved in the taxol biosynthesis pathways were analyzed. As described above, the rate-limited genes involved in supply of terpenoid precursor and C13-side chain were significantly up-regulated at 0.5 h after KL27-FB treatment (Fig. 4b). Then, the genes involved in diterpenoid taxane core biosynthesis and baccatin III formation steps of taxol biosynthesis were also analyzed. Our RNA-seq results showed that most of the defined unigenes involved in taxol biosynthesis were up-regulated at 0.5 h after KL27-FB elicitation, whereas, the expression of more than half of them decreased at 6 h after KL27-FB elicitation. In detail, for the diterpenoid taxane core biosynthesis, the genes encoding the key enzymes, *GGPPS* and *TS*, predominantly expressed at 0.5 h after KL27-FB treatment (Fig. 4b), while the expression of TS-encoding genes decreased at 6 h after KL27-FB treatment. For the baccatin III formation step, a serious of unigenes corresponding to almost all of the enzymes involved were increased obviously at 0.5 h after KL27-FB treatment. But at 6 h after KL27-FB treatment, the expression of more than half of these genes decreased. These results indicated that KL27-FB could promote the taxol accumulation by up-regulating of the enzymes of the upper and down streams of taxol biosynthesis pathway. And the activation of the taxol biosynthesis pathway by KL27-FB occurred in the early stage of stimulation, and decreased with the passage of time.

Additionally, the expression levels of six unigenes involved in taxol biosynthesis were random selected and verified by qRT-PCR. Our results indicated that, qRT-PCR expression profiles of the five genes were found to be consistent with the RNA-seq data at both 0.5 h and 6 h after KL27-FB treatments (Fig. 4c and d and Additional file 10). These results further indicated the reliability of the RNA-seq data.

Interestingly, we found several identified unigenes, which were corresponding to hydroxylase, epoxidase, oxomutase, oxidase and β-phenylalanine CoA ligase, which provided candidates of presumptive enzymes for the remaining steps of taxol biosynthesis pathways, such as C1 β-hydroxylase, C9 α-hydroxylase, C4, C20-epoxidase, pyridine nucleotide-dependent dehydrogenase, and β-phenylalanoyl CoA ligase (Additional file 11).

### KL27-FB effected hormone biosynthesis and signal transduction

Phytohormone plays important roles in plant growth, development and stress responses. And the plant hormone signal transduction pathway is also been shown to affect taxol biosynthesis in *Taxus*. Our RNA-seq data showed that eight and twenty-five hormone-related GO terms involved in auxin, jasmonic acid (JA), gibberellin acid (GA), ethylene (ET), salicylic acid (SA), cytokinin (CTY), abscisic acid (ABA) and brassinosteroid (BR) were enriched (*p* < 0.05) at 0.5 h and 6 h after KL27-FB treatment respectively (Fig. 5b). Which indicated that KL27-FB could significantly effect the hormone metabolism and transduction in *T.chinensis* needles.

Tryptophan metabolism, zeatin biosynthesis, diterpenoid biosynthesis, caroternoid biosynthesis, cysteine and methionine metabolism, brassinosteroid biosynthesis, α-linolenic acid metabolism and phenylalanine metabolism pathways were in response to the biosynthesis of auxin, CTY, GA, ABA, ET, BR, JA and SA, respectively. Our results showed that, after KL27-FB treatment, these genes encoding for amidase (amiE) and indole-3-pyruvate monooxygenase (YUCCA) in the biosynthesis of auxin, genes corresponding to steroid 22-alpha-hydroxylase (DWF4) and PHYB activation tagged suppressor 1 (BAS1) in BR biosynthesis pathway, genes encoding for 12-oxophytodienoic acid reductase (OPR) and jasmonate O-methyltransferase (JMT) in JA biosynthesis showed increased transcript abundance. For TYC synthesis, the gene encoding for cytokinin trans-hydroxylase (CYP735A) in TYC biosynthesis was increased and the gene-encoding for cytokinin dehydrogenase (CKX) in TYC peroxidative degradation is decreased after KL27 treatment. These results implied the synthesis of auxin, CTK, JA and BR were activated after KL27-FB stimulation. In contrast, genes encoding for 9-cis-epoxycarotenoid dioxygenase (NCED) a rate-limited enzyme in the ABA syntheses and (+)-abscisic acid 8′-hydroxylase (ABA8ox) in ABA oxidative inactivation were decreased. Genes corresponding to ent-copalyl diphosphate synthase (GPS), gibberellin 3 beta-dioxygenase (GA3ox), ent-kaurene synthase (KS) and ent-kaurenoic acid monooxygenase (KAO) in the biosynthesis of GA and gene corresponding to 1-aminocyclopropane-1-carboxylate oxidase (ACO) in the biosynthesis of ET, displayed decreased transcript abundance after KL27-FB treatment, which implied represses in ABA, GA and ET biosynthesis after KL27-FB elicitation.

Furthermore, based on the KEGG analysis, “plant hormone signal transduction” (ko04075) were significantly enriched after KL27-FB treatment (Fig. 3f). Thirty-seven and fourty-five significant DEGs were enriched in “plant hormone signal transduction” (ko04075) at 0.5 h and 6 h after KL27-FB treatments respectively, These unigenes are mainly enriched in auxin, CTY, JA, GA, ABA, ET, BR and SA signal transductions. For auxin signaling, the expression of genes corresponding to auxin-responsive protein IAA (AUX/IAA), auxin responsive GH3 gene family (GH3) and some of SAUR family proteins (SAUR) were highly up-regulated after KL27-FB treatment, while auxin influx carrier 1 (AUX1) was decreasing expressed in the auxin signaling pathway at 6 h after KL27-FB treatment. Genes encoding for cytokinin receptor 1 (CRE1) and two-component response regulator ARR-B family (B-ARR) were kept down-regulated after KL27-FB treatment over time, while two-component response regulator ARR-A family (A-ARR) was significantly decreasing expressed in the cytokinine signaling pathway at 0.5 h after KL27-FB treatment. For ABA signaling transduction, the expression of genes corresponding to serine/threonine-protein kinase SRK2 (SnRK2) and ABA responsive element binding factor (ABF) were down-regulated after KL27-FB treatment over time. While, abscisic acid receptor PYR/PYL family (PYL)-encoding gene and serine/threonine-protein phosphatase 2A catalytic subunit (PP2C) was up-regulated at 6 h after KL27-FB treatment. For BR signaling transduction, genes encoding for BR-signaling kinase (BSK) and xyloglucan:xyloglucosyl transferase TCH4 (TCH4) were up-regulated after KL27-FB treatment. Genes corresponding to jasmonate ZIM domain-containing protein (JAZ) and MYC2 had significantly increased transcript abundance at 0.5 h after KL27-FB treatment, while the gene encoding for coronatine-insensitive protein 1 (COI-1) and some of JAZs showed highly up-regulation in the JA signaling pathway at 6 h after KL27-FB treatment. In SA signaling pathway, genes corresponding to basic salivary proline-rich protein 1 (PR1) showed down-regulation after KL27-FB treatment, while nonexpresser of pathogenesis-related gene 1 (NPR1)-encoding gene showed up-regulation at 0.5 h and down-regulation at 6 h after KL27-FB elicitation. In the GA signaling pathway, genes encoding for gibberellin receptor GID1 (GID1) and DELLA were significantly up-regulated at 6 h after KL27-FB treatment, which genes encoding for F-box protein GID2 (GID2) were both significantly down-regulated at 0.5 h and 6 h after KL27-FB treatments. For the ET signaling pathway, the unigene encoding for the ethylene receptor (ETR) was significantly up-regulated after KL27-FB treatment. While, the ERF2 TF encoding gene was up-regulated at 0.5 h after KL27-FB treatment. Furthermore, most of these DEGs were involved in plant cell growth and defense response (Additional file 12). These results indicated that, after KL27-FB treatment, the signal transduction pathways of auxin, ET and JA were activated, while the signal transduction pathways of CYT, ABA, BR and SA showed repressed at 0.5 h after the elicitation. And the signal transduction pathways of CTY, ET and BR did not change significantly at 6 h after the elicitation. However, compared to the 0.5 h, the Auxin, ABA, JA, GA and SA signal transduction showed variation at 6 h after KL27-FB elicitation. These results suggested that *T. chinensis* cells replied the KL27-FB elicitor through the complex hormone signal pathways, and these hormone levels changed dynamically over time after the KL27-FB stimulation. Thus changed the plant growth and the stress response pathways .

### Regulation of the expression of TFs in *T. chinensis* after KL27-FB treatment

A great number of TFs were reported to play important roles in taxol biosynthesis. In this study, 1068 putative TF encoding genes belonging to 67 major TF families were identified in *T. chinensis* (Additional file 13). These TFs were largely belonged to families such as the MYB (Myble) superfamily (134 unigenes), AP2/ERF superfamily (109 unigenes), C2H2 supfamily (66 unigenes) and bHLH (66 unigenes). The number of different expressed TFs after KL27-FB treatments were shown in Additional file 13. Among these TFs, 183 DEGs including 108 up-regulated and 75 down-regulated were identified at 0.5 h after KL27-FB treatment, and 291 DEGs including 162 up-regulated and 129 down-regulated were identified at 6 h after KL27-FB treatment. These DEGs analysis revealed that most of the TFs were significantly up-regulated after KL27-FB treatment. To identify key regulators for taxol biosynthesis, the change of the expression levels of these TF families, which have been reported to regulate taxol biosynthesis in *Taxus* including AP2/ERF, MYB, WRKY and bHLH families [39–45] were shown in a heatmap (Additional file 14). DEGs analysis revealed that most of these TFs were highly up-regulated after KL27-FB treatment. Some of these TFs keep their expression pattern at 0.5 h and 6 h after elicitation. However, most of these TF-encoding genes have opposite intensity of expression at 0.5 h and 6 h after elicitation. Furthermore, The DEGs encoding for TFs after KL27-FB treatment were mainly associated with the regulation of the secondary metabolites and the defense response.

## Discussions

Plant endophytic microbes, including bacterias, fungi and actinomycetes, parasitize in plants but don’t cause plant diseases, which are essential for the growth and development of their host plants. Among them, endophytic fungus are formed by a very diverse group of microorganisms, and almost all plants contain endophyte fungus. These fungi can generate various chemical compounds, which will induce complex metabolic changes of their host plants, especially in the promoting of biosynthesis and accumulation of secondary metabolites [46]. For example, oligosaccharide elicitor of endophytic fungus *Fusarium oxysporum* Dzf17, isolated from *Dioscorea zingiberensis* could enhance the diosgenin production in *D. zingiberensis* cell cultures [19]. Chen H.M. et al. [47] isolated an endophytic fungus, *Mucor circinelloides* DF20, from *S. miltiorrhiza* roots could significantly increase the tanshinone biosynthesis and accumulation in *S. miltiorrhiza* roots. Jie Y. et al. [48] indicated that endophytic fungus *Gilmaniella* sp. AL12 in *Atractylodes lancea* could stimulate the sesquiterpenids biosynthesis via inducing ethylene production in *A. lancea.*

*P. lobariellae* is a lichenicolous specie sister to saprobic genus *Kalmusia.* It is a new genus placed in *Didymosphaeriaeae* and firstly isolated from *Lobariella* in Bolivia in 2019 [49]. Till now, there is no report about its metabolites and its effect on host metabolisms. However, in this study, our reaserches indicated that the fermentation broth of endophytic fungus *P. lobariellae* KL27 could promote the conversion of the precursors to end products of taxol biosynthesis pathway, resulting in taxol accumulation in *T.chinensis* needles. As shown in Fig. 1, after treatment with the KL27-FB, the content of taxol in *T.chinensis* needles reached from 0.111 ± 0.050 mg/g·DW to 0.361 ± 0.082 mg/g·DW. By RNA-sequencing analysis of the key enzyme genes of taxol biosynthesis pathway caused by KL27-FB treatment, we found most of the genes of taxol biosynthesis pathway were up-regulated after 0.5 h of KL27-FB treatment, but subsequently declined after 6 h of the stimuli, except for *T5OH* (DN13577_c0g1i1.2), *TAT* (DN24472_c0g2i4.1) *T10OH* (DN23758_c2g6i1.2 and DN23394_c1g1i2.2), *T2OH* (DN23627_c0g1i1.2), *TBT* (DN22572_c0g1i1.2), *PAM* (DN22851_c0g1i1.2) and *BAPT* (DN24543_c0g1i1.2 and DN24543_c1g1i3.1) (Fig. 4b). These results indicated that KL27-FB could significantly promoted the taxol biosynthesis of *T.chinensis* needles, and the promoting effect reduced over time.

However, taxol is not the main metabolite, for a very large number of side-chain variants differ in the position of the hydroxylated taxane nucleus as well as the type of acyl/aroyl substitution have been isolated in the *Taxus* sp., resulting in over 350 taxane diterpenoids. And these side reactions greatly affect the yield of taxol production [50, 51]. In this study, the KL27-FB treatment of the needles of *T. chinensis*, could improved the accumulation of the final content taxol from the precursors, however the decrease of precursor (baccatin III and 10-DAB) contents were much more than the increase of end product, indicated that side routes produced by acyl/aroyl or the oxidation of the taxane nucleus derived from common precursors can compete with taxol biosynthesis (Fig. 1). Identification of these side-route genes could have an important implication in eventually increasing of taxol yields.

JA and its derivative MeJA, are stress hormones which can induce the biosynthesis of some secondary metabolites. Many studies have shown that MeJA can induce terpene accumulate in conifers [52]. And MeJA is also one of the most effective inducers of taxol biosynthesis in taxol cell cultures [53]. Yukimune, Y. et al. [40] found that exogenously adding of MeJA could induce the production of taxol in *Taxus* cell suspension cultures. Furthermore, increasing evidences showed that MeJA-mediated transcriptional regulation of secondary pathways is likely to be orchestrated by the action of multiplex TFs such as WRKY, bHLH and AP2/ERF. Combinatorial action of bHLH and AP2/ERF factors has already been shown in the JA-induced responses of nicotine and alkaloid biosynthesis [41]. Other classes of MeJA-responsive TFs such as WRKYs and MYBs also have been shown to regulate JA mediated responses [42–45, 54, 55]*.* Sangram K et al. [55] isolated three MeJA-regulated bHLH TFs in *T. cuspidata*, and indicated that these TFs actived as negative regulators of MeJA-mediated expression of taxane biosynthetic genes in *Taxus* cell cultures. Zhang M et al. [54] identified two JA-responsive factors, TcERF12 and TcERF15, which acted as negative and positive regulators of *tasy* gene of taxol biosynthesis in *T. chinensis* respectively. In this study, a number of DEGs associated with JA synthesis and signal pathways were identified, suggesting variants in JA biosynthesis and signaling after KL27-FB treatment. The increased transcript aboundances of genes *AOS, OPR* and *JMR* in JA biosynthesis process at the begin stage (0.5 h) after KL27-treatment, suggested a higher JA level in *T. chinensis*, Then these synthetic JA medicated the binding of COI1 to JAZ, which made the degradation of the complex by 26S proteasome and frees MYC2, which in turn acted in the regulation of the expression of JA-inducting genes [56, 57]. As time went on, JA level was decreased by the down-regulated expression of JA biosynthesis genes such as *AOS* and *JMT*, and the JA signal transduction decreased with the highly expressed *JAZs* genes, resulting in re-estabilishing of binding between MYC2 and JAZs, which blocked the MYCs transcriptional regulatory activity, and stopped the regulation of the expression of some JA-inducting genes. These results may explain most of the differential expression of genes involved in taxol biosynthesis pathway after KL27-FB treatment over time (Fig. 4b). All these results revealed that JA signal may acted in the transmission of KL27-FB stimuli signal and affected the taxol biosynthesis in needles of *T. chinensis*. These genes involved in the response after KL27-FB elicitor are worthy for further study in the future.

Increased evidence shows that the JA signal pathway has crosstalk with other hormone transduction pathways in the secondary metabolisms biosynthesis, such as GA, ET and SA signaling. DELLA protein, which has a similar role with JAZs, plays a key negative regulatory role in the GA signal transdution. In the presence of F-box SLY1 (or GID2) and GA, DELLA interacting with GID1 and activated GA-respondent genes via degradation the DELLA-GA-GID1 by the 26S proteasome. The increase expression of the GID1 gene and DELLA gene and decrease expression of GID2 in RNA-seq analysis at 6 h after KL27-FB treatment, indicated that KL27-FB could activate GA signaling pathway, and its interaction with the JA pathway. DELLA can interacts with JAZ [56], acted in the release of TFs including PIF and MYC2 [57] to regulate the balance of JA and GA signaling. Which may promote the binding of DELLA protein with JAZ, and help for the free of MYC2. The decrease of a series of genes related to GA biosynthesis implied a decrease in the GA level after KL27-FB treatment, which was inconsistent with the active GA singal transduction. However, various studies indicated that fungus can product GA. So KL27-FB may contain GA, which could improve the GA level in *T. chinensis* cells. Therefore, mutual promotion between JA and GA after KL27-FB treatment could be a means for regulating development and the defense response. In general, JA and SA are two important defense-related hormones, which work against each other in plant immune signaling. In this study, at the begin stage after KL27-FB elicitation, SA signaling pathway was significantly inhibited through the decreased of some key genes encoding for NPR1, TGA, and PR1; while at the end stage after KL27-FB stimuli, SA signaling pathway was activated through the increased expression of key genes encoding for NPR1 (DN34244_c0g1i1.2). Which indicated that GA and SA signal are antagonistic during the responses of *T. chinensis* cell to KL27-FB stimuli. Moreover, a serious of ET-biosynthesis genes (cysteine and methionne metabolism) are down-regulated, suggested a decrease in ET level after KL27-FB treatment, which indicated ET signal may be involving in negatively regulation of taxol biosynthesis. The up-regulation of ERF1/2 genes after KL27-FB treatment may be related to JAZ in JA signaling [54]. Furthermore, the active CTY and auxin biosynthesis, suggested the promote effect of the fungus in the growth of *T.chinensis,* which are sure to be worthy for further studying. The synergistic effects between phytohormones such as JA, GA, SA and ethylene after KL27-FB treatment further indicates a specific regulatory pattern between plant development and defense after KL27-FB stimuli. Due to the complexity of multiple signal interaction networks, independent validation is still necessary to accurately measure the level of expression of interested genes.

**Fig. 6:**
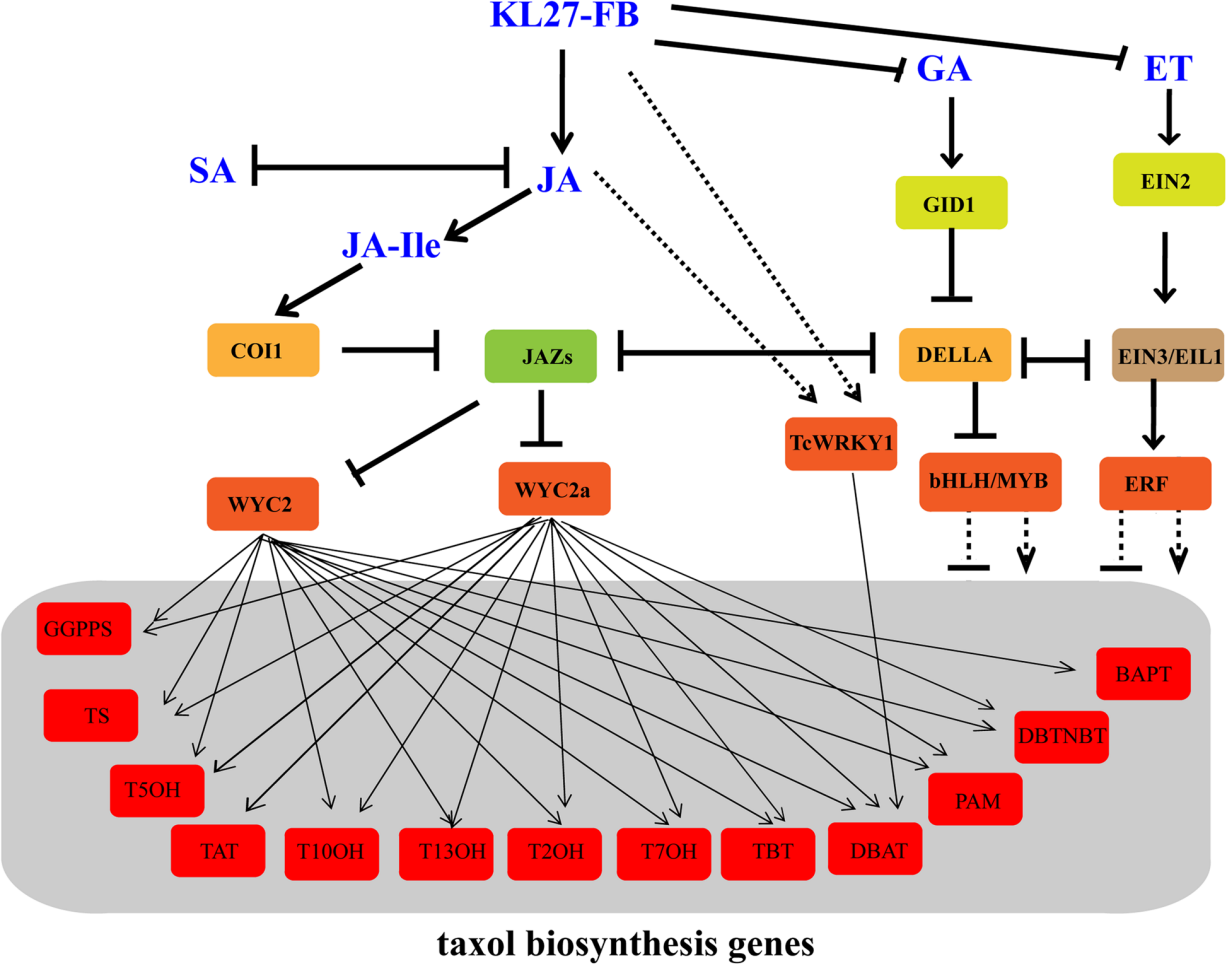
Schematic diagram of the JA signaling-medicated response to KL27-FB elicitation and its regulation of the taxol biosynthesis pathway. The solid arrow indicates that are directly activated the protein or up-regulated the gene expression, The dotted arrow indicates that are indirectly up-regulated the genes expression, ‘┤’ indicates that are directly represses the protein function or gene expression

Regulation of biosynthesis genes via TFs is one of the major regulatory mechanism of secondary metabolite production in plant cells [58]. A serious of TFs have been evaluated to regulated the expression of genes in taxol biosynthesis pathway. Our RNA-seq results also showed that many genes encoding TFs, including MYC, WRKY, bHLH and AP2/ERF were differently expressed after KL27-FB elicitation, which may be directly or indirectly regulate the expression of genes involved in taxol biosynthesis. Le et al. [43] reported that TcWRKY1 have a positive regulatory role on the expression of *DBAT* gene, which show a similar expression pattern with *DBAT* at 0.5 h after KL27-FB*.* And the expression of *TcWRKY1* was highly induced by both MeJA and fungal elicitor F5, suggested that KL27-FB and fungal elicitor F5 improved the taxol biosynthesis with a similar regulatory pathway in which MeJA signal was also involved. WYC2 is one of the most important regulator in the JA signal transfer and secondary metabolism biosynthesis in plant. Zhang et al. [59] isolated a TcMYC2a, which can regulate the taxol biosynthesis directly by positive regulate the expression of taxol biosythesis genes including *TS*, *TAT*, *DBTNBT*, *T13OH*, *T50H*, or indirectly via positive regulate of ERF15 depending on JA signal transduction. Our RNA-seq data showed the MYC2a (DN24851_c0g4i3.2) was 1.68-fold up-regulated at 0.5 h after KL27-FB treatment, and decreased to normal status at 6 h after KL27-FB treatment. Which show a similar expression pattern with *TS*, *TAT*, *DBTNBT*, *T13OH*, and some unigenes corresponding to *T5OH*. Unfortunately, our transcriptome date did not mapped unigene corresponding to ERF15. Cui et al. [60] reported their studies on the regulation mechanism of MYC family in JA signal pathway on taxol biosynthesis. According to their studies, MYC2, MYC3 and MYC4 could activate 12, 10 and 11 taxol biosynthesis genes promoters respectively (Additional file 15). We mapped our RNA-seq data with the MYC family. Unigene DN22125_c0gi4.1 (Nr annation: JAMYC2), DN1651_c0g1i1.2 and DN24851_c0g4i3.2 (Nr annation: MYC2a) have high identify (98%) with MYC2, MYC3 and MYC4 respectively. Our RNA-seq data showed that the MYC2 and MYC2a was 1.37- and 1.68 up-regulated at 0.5 h after KL27-FB treatment, and decreased to 0.71- and 0.83-fold down-regulated at 6 h after KL27-FB treatment. While JAMYC2 have no differential expressed after KL27-FB treatment. And the expression pattern of all of these unigenes involving in taxol biosynthesis, excepted for four unigenes including DN23243_c0g1i2.2 (*GGPPS*) and DN24734_c0g1i2.1 (*PAM*) (Fig. 4b) were consistent with the MYCs. Moreover, in this study TcERF12 showed up-regulated after KL27-FB treatment (Additional file 13), while Zhang et al. [54] reported the negative regulation of a JA-responsive factor TcERF12 on its target gene *TS* in *T. chinensis*, which was inconsistent with our study. And, there were still some genes kept highly expressed at 6 h after KL27-FB treatment which was inconsistent with the decreased expression of MYC2s and TcWRKY1. The reasons may be due to the complex regulatory network of genes in taxol biosynthesis. Therefore, one major regulatory mechanism of increasing taxol biosynthesis after KL27-FB elicitation was via controling of the expression of TFs, which was associated to the crosstalk between JA and other hormonal signals (Fig. 6). Moreover, most of these differential expressed TFs after KL27-FB treatment were involved in cell growth and defense responses. These results suggested that many genes encoding TFs may act regulate roles in the plant growth and development, as well as stress response, and regulate the expression and activity of enzymes in taxol biosynthesis directly or indirectly. Thus, characterization of these TFs might shed some light on the molecular mechanism regulation of taxol biosynthesis in *Taxus*.

However, the results of these study were obtained from the treatment of the fermentation broth of KL27 on the needles of *T.chinensis*. It needs for further study about the influence of co-incubation of the KL27 on the growth and secondary metabolism of *T.chinensis*. Moreover, the mechanisms of signal transduction pathway which medicate some of the enzymes expression involved in the taxol biosynthesis after KL27-FB elicitation is still unclear, and the related effector of KL27-FB and its action targets on the *T.chinensis* needles are sure to be worthy studying.

## Conclusions

Up-regulating the expression of the taxol biosynthesis pathway-related genes, involving in precursor supply, diterpenoid taxane core-syntheisis, bacctin III formation and C13-side chain assembly might provide a potential explanation for the accumulation of taxol in needles of *T.chinensis* after endophytic fungus treatment. JA signal and its crosstalk with phytohormone (eg: GA and ET) were involved in the signal transduction process of endophytic fungus stimuli, and regulate the expression of taxol-biosynthesis genes, resulting the accumulation of taxol. Our studys contribute to a deeper understanding of the accumulation of taxol in *T.chinensis* needles after fermentation broth of endophytic fungus, *P. lobariellae KL27* treatment.

## Supplementary Information

**Additional file 1: Table S1**. Primes of selected target genes for qRT-PCR.

**Additional file 2: Table S2**. The detail information of raw reads from different sample groups. (XLS 21 kb)

**Additional file 3: Figure S1**. Principal components analysis of the four transcriptomes.

**Additional file 4.** Assembled unigenes in this study.

**Additional file 5: Figure S2**. GO and KEGG annotation and KOG category classification of all unigenes.

**Additional file 6: Figure S3**. GO classification of DEGs at 0.5 h (**a**) and 6 h after KL27-FB treatment (**b**).

**Additional file 7: Table S3**. GO and KEGG enrichment analysis. (XLS 20 kb)

**Additional file 8: Table S4**. DEGs involving in KEGG pathways after KL27-FB treatment. (XLS 202 kb)

**Additional file 9: Table S5**. Differential expression of all unigenes in phenylpropanoid biosynthesis pathway (ko00940). (XLS 81 kb)

**Additional file 10: Table S6**. Differential expression of random selected genes.

**Additional file 11: Table S7**. Annotation of unigenes. (XLS 8884 kb)

**Additional file 12: Figure S4**. Hormone metabolism and signal transduction of auxin (a), CTY (b), ABA (c), ET (d), BR (e), JA (f), SA (g) and GA (h) after KL27-FB treatment.

**Additional file 13: Table S7**. Annotation to encode putative TFs. (XLS 943 kb)

**Additional file 14: Figure S5**. The expression change of each TF-encoding genes in the two comparisons was shown by a heatmap.

**Additional file 15: Table S8**. MYC2, MYC3 and MYC4 regulate effect on the taxol biosynthesis genes.

## Data Availability

The datasets generated and analysed during the current study are available in the NCBI Short Read Archive with accession number PRJNA751266.

## Abbreviations

KL27-FB: fermentation broth of KL27
DW: dry weight
RNA-Seq: RNA sequencing
Nr: nonredundant protein sequence database
eggNOG: non-supervised orthologous groups
KOG: clusters of orthologous groups for eukaryotic complete genomes
FPKM: fragments per Kilobase per Million
FDR: false discovery rate
CK0.5H: the controls at 0.5 h after PDB treatment
Y0.5H: samples at 0.5 h after K27-FB treatment
CK6H: the controls at 6 h after PDB treatment
Y6H: samples at 6 h after K27-FB treatment
TF: transcription factor
MeJA: methyl jasmonic acid
DXS: 1-deoxy-D-xylulose 5-phosphate synthase
DXR: 1-deoxy-D-xylulose 5-phosphate reductoisomerase
HDR: 4-hydroxy-3-methylbut-2-enyl diphosphate reductase
GGPPS: geranylgeranyl diphosphate synthase
TS: taxadiene synthase
T5OH: taxane 5a-hydroxylase
TAT: taxadiene-5α-ol-O-acetyl transferase
T10OH: taxane 10β-hydroxylase
T13OH: taxane 13α-hydroxylase
T2OH: taxane 2α-hydroxylase
T7OH: taxane 7β-hydroxylase
T14OH: taxane 14β-hydroxylase
TBT: taxane-2α-O-benzoyltransferase
DBAT: 10-deacetyl baccatin III-10-O-acetyltransferase
PAM: phenylalanine aminomutase
BAPT: 3-amino-3-phenylpropanoyltrans-ferase
DBTNBT: 3'-N-debenzoyltaxol N-benzoyltransferase
PAL: phenylalanine ammonialyase
GGPP: geranylgeranyl diphosphate
IPP: isopentenyl diphosphate
DMAPP: dimethylallyl diphosphate
MEP: 2-C-methyl-D-erythritol 4-phosphate pathway
DEG: differential expressed unigenes
GO: gene ontology
JA: jasmonic acid
KEGG: kyoto encyclopedia of genes and genomes
GA: gibberellin acid
ET: ethylene
SA: salicylic acid
ERFs: ethylene-responsive transcription factors
bHLH: basic/helix-loop-helix
qRT-PCR: quantitative Real-Time PCR
4CL: 4-coumarate CoA ligase
COMT: caffeic acid 3-O-methyltransferase
HCT: shikimate O-hydroxy cinnamoyltransferase
C3'H: *p*-coumarate 3-hydroxylase
ispD: 2-C-methyl-D-erythritol 4-phosphate cytidylyltransferase
ispE: 4-diphosphocytidyl-2-C-methyl-D-erythritol kinase
ispF: 2-C-methyl-D-erythritol 2,4-cyclodiphosphate synthase
ispG: (E)-4-hydroxy-3-methylbut-2-enyl-diphosphate synthase
ispH: 4-hydroxy-3-methylbut-2-en-1-yl diphosphate reductase
idi: isopentenyl-diphosphate Delta-isomerase
CTY: cytokinin
ABA: abscisic acid
BR: brassinosteroid
amiE: amidase
YUCCA: indole-3-pyruvate monooxygenase
DWF4: steroid 22-alpha-hydroxylase
BAS1: PHYB activation tagged suppressor 1
OPR: 12-oxophytodienoic acid reductase
JMT: jasmonate O-methyltransferase
CYP735A: cytokinin trans-hydroxylase
CKX: cytokinin dehydrogenase
NCED: 9-cis-epoxycarotenoid dioxygenase
ABA8ox: (+)-abscisic acid 8'-hydroxylase
GPS: ent-copalyl diphosphate synthase
GA3ox: gibberellin 3 beta-dioxygenase
KS: ent-kaurene synthase
KAO: ent-kaurenoic acid monooxygenase
ACO: 1-aminocyclopropane-1-carboxylate oxidase
AUX/IAA: auxin-responsive protein IAA
GH3: auxin responsive GH3 gene family
SAUR: SAUR family proteins
AUX1: auxin influx carrier 1
CRE1: cytokinin receptor 1
B-ARR: two-component response regulator ARR-B family
A-ARR: two-component response regulator ARR-A family
SnRK2: serine/threonine-protein kinase SRK2
ABF: ABA responsive element binding factor
PYL: abscisic acid receptor PYR/PYL family
PP2C: serine/threonine-protein phosphatase 2A catalytic subunit
BSK: BR-signaling kinase
TCH4: xyloglucan:xyloglucosyl transferase TCH4
JAZ: jasmonate ZIM domain-containing protein
COI-1: coronatine-insensitive protein 1
PR1: basic salivary proline-rich protein 1
NPR1: nonexpresser of pathogenesis-related gene 1
GID1: gibberellin receptor GID1
GID2: F-box protein GID2
