# Supplementary material for: Endophytic fungus Pseudodidymocyrtis lobariellae KL27 promotes taxol biosynthesis and accumulation in Taxus chinensis
Source: BMC Plant Biol. 2022 Jan 3;22:12. doi: 10.1186/s12870-021-03396-6 (PMC8722197; doi:10.1186/s12870-021-03396-6)
Supplement: Supplementary file 1 — Additional file 1: Table S1. Primes of selected target genes for qRT-PCR. [file 12870_2021_3396_MOESM1_ESM.docx]

Table S1 Primes of selected target genes for qRT-PCR.

| Name | Gene-ID | Primer Sequences |
| --- | --- | --- |
| GGPPS | DN23243_c0g3i1.2 | F: CCGGTGTGTGGGGCTTCTGTTT  R: TCGTCGCCAATTCAGCGGCA |
| TS | DN24279_c0g1i23.2 | F: GGGCATCGGTTGGGGCAGAG  R: TCGACATGGGTTTGGCCCGC |
| T2OH | DN23627_c0g1i1.2 | F: CGCAGCCGGAAACCGCCTAA  R: TGCCGGCCCCAAAAACCCTG |
| T5OH | DN25136_c0g2i3.1 | F: GCAGATGTCGTGGCCCGCTC  R: AGCGCACCAGGGCCGAAAAA |
| T7OH | DN26002_c0g2i1.1 | F: GCCCTGCGGGAAACCGCTTA  R: CGACACGCAGAACGCGGTGA |
| GAPDH | DN19780_c0g1i3.2 | F: CGGAGACAGTCGATCAAGC  R: CCCATCCTCAACCCAATAA |
|  |  |  |
