# Supplementary figures and images for: Endophytic fungus Pseudodidymocyrtis lobariellae KL27 promotes taxol biosynthesis and accumulation in Taxus chinensis
Source: BMC Plant Biol. 2022 Jan 3;22:12. doi: 10.1186/s12870-021-03396-6 (PMC8722197; doi:10.1186/s12870-021-03396-6)

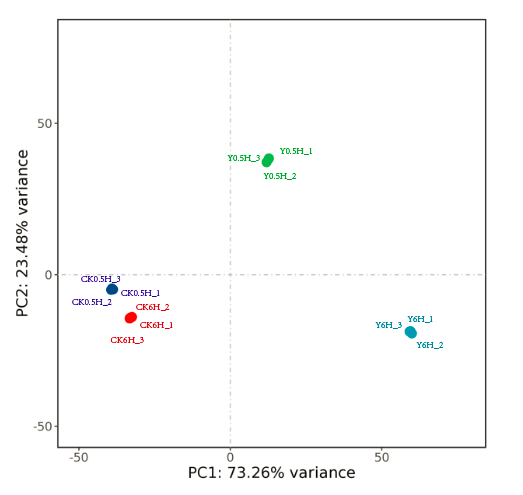


Figure S1 Principal components analysis of the four transcriptomes.

Supplement: Supplementary file 3 — Additional file 3: Figure S1. Principal components analysis of the four transcriptomes. [file 12870_2021_3396_MOESM3_ESM.doc]

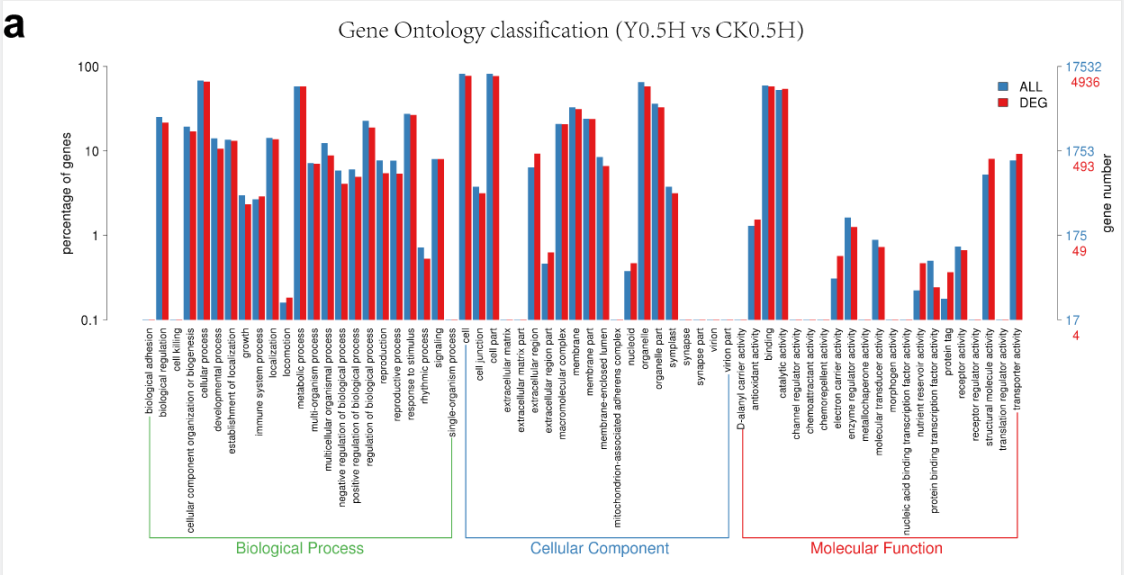


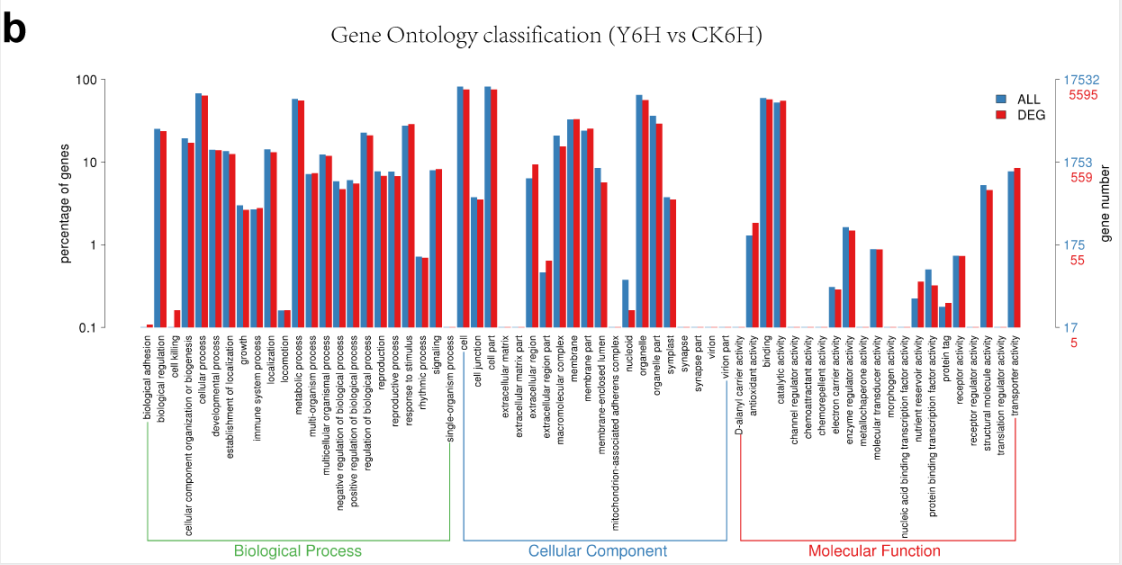


Figure S3 GO classification of DEGs at 0.5 h (**a**) and 6 h after KL27-FB treatment (**b**).

Supplement: Supplementary file 6 — Additional file 6: Figure S3. GO classification of DEGs at 0.5 h (a) and 6 h after KL27-FB treatment (b). [file 12870_2021_3396_MOESM6_ESM.doc]
