# Supplementary material for: Endophytic fungus Pseudodidymocyrtis lobariellae KL27 promotes taxol biosynthesis and accumulation in Taxus chinensis
Source: BMC Plant Biol. 2022 Jan 3;22:12. doi: 10.1186/s12870-021-03396-6 (PMC8722197; doi:10.1186/s12870-021-03396-6)
Supplement: Supplementary file 5 — Additional file 5: Figure S2. GO and KEGG annotation and KOG category classification of all unigenes. [file 12870_2021_3396_MOESM5_ESM.doc]

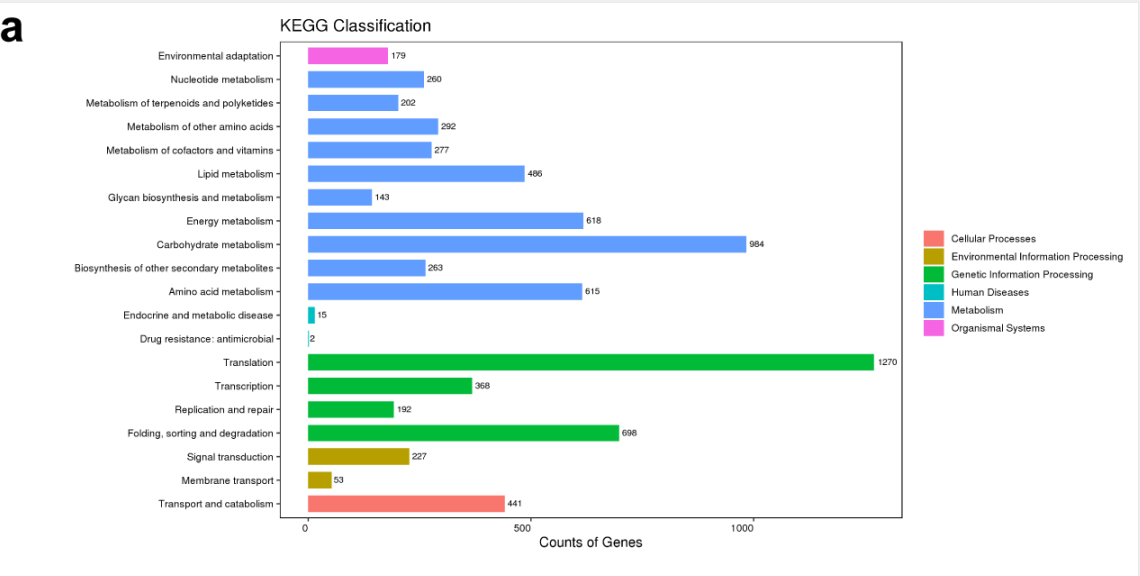


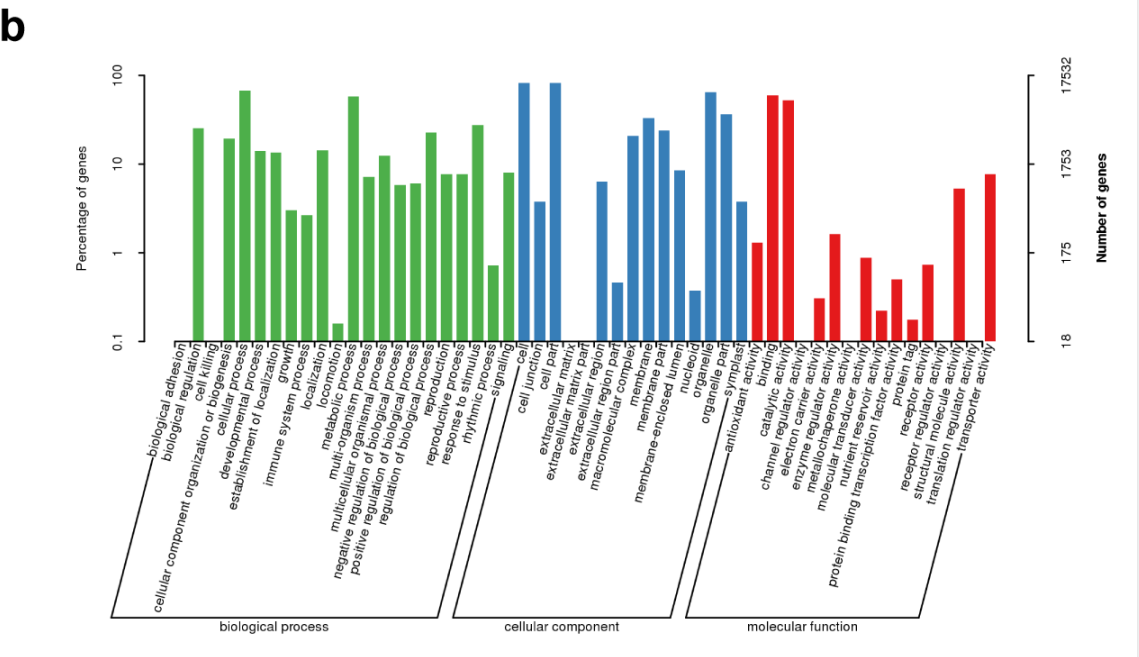


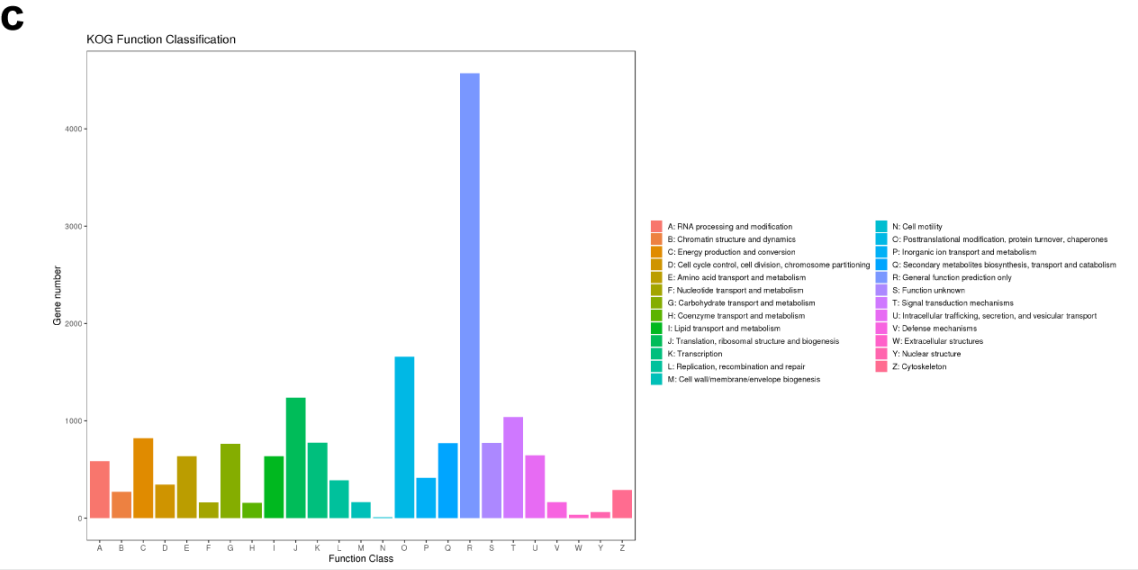


Figure S2. GO and KEGG annotation and KOG category classification of all unigenes. **a** KEGG annotation of all unigenes. **b** GO annotation of all unigenes. Annotated sequences were classified into “biological process”,“molecular function”, and cellular component” groups and 53 subgroups. **c** KOG category classification of all unigenes.
