# Supplementary material for: Endophytic fungus Pseudodidymocyrtis lobariellae KL27 promotes taxol biosynthesis and accumulation in Taxus chinensis
Source: BMC Plant Biol. 2022 Jan 3;22:12. doi: 10.1186/s12870-021-03396-6 (PMC8722197; doi:10.1186/s12870-021-03396-6)
Supplement: Supplementary file 12 — Additional file 12: Figure S4. Hormone metabolism and signal transduction of auxin (a), CTY (b), ABA (c), ET (d), BR (e), JA (f), SA (g) and GA (h) after KL27-FB treatment. [file 12870_2021_3396_MOESM12_ESM.doc]

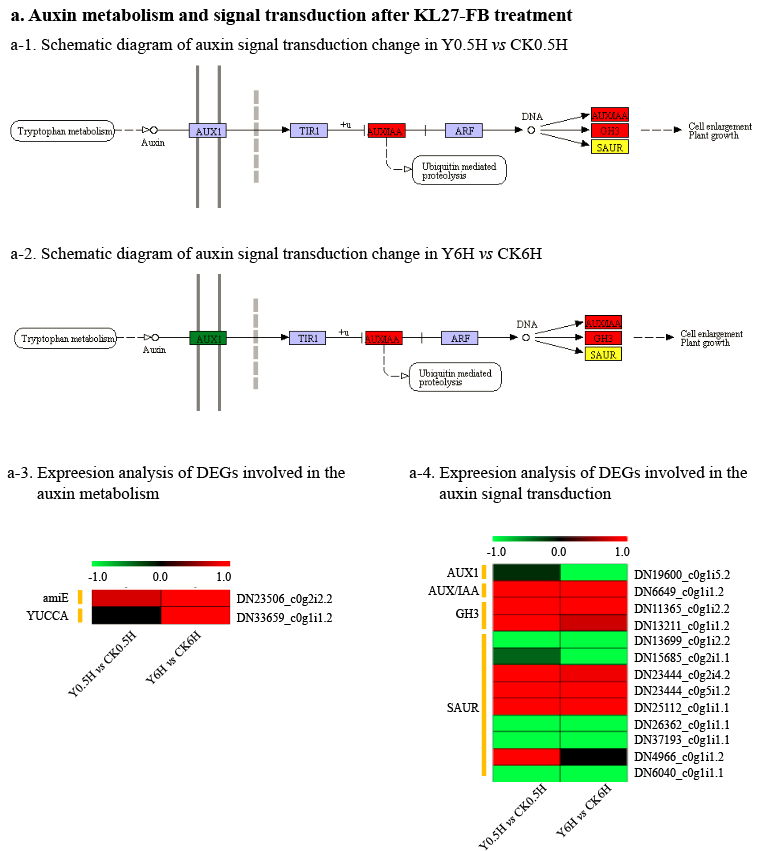

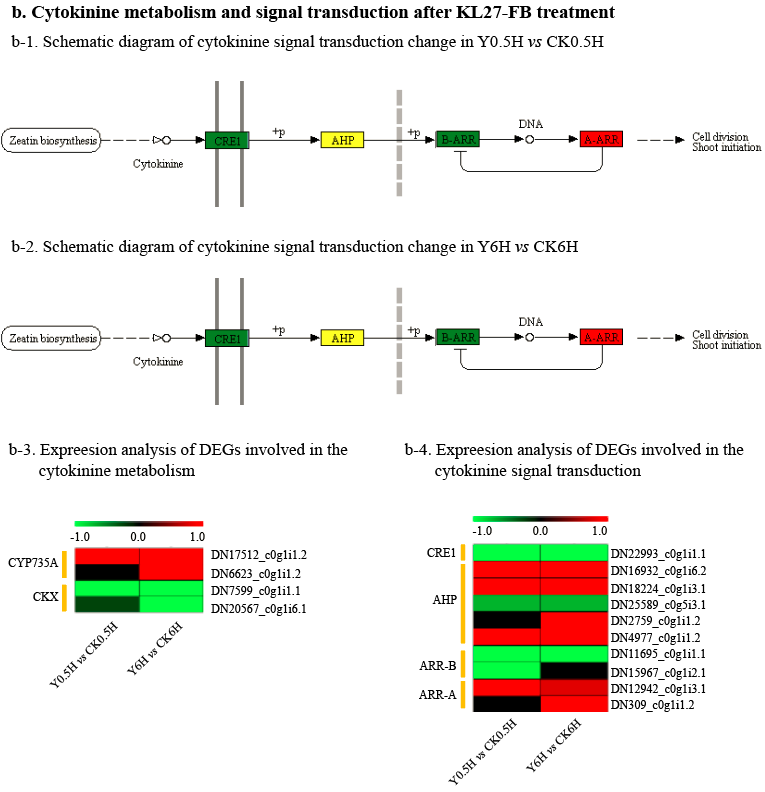

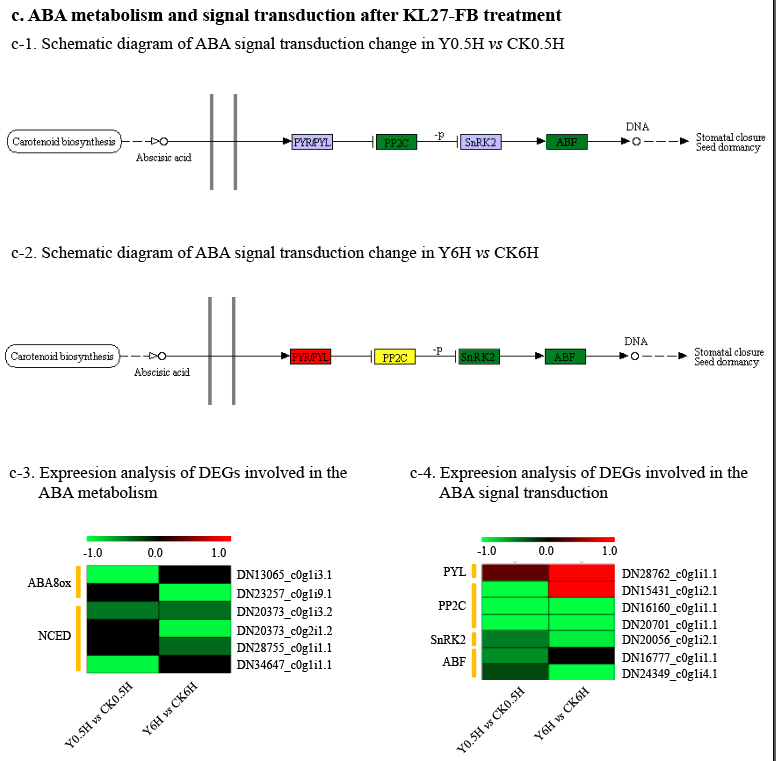

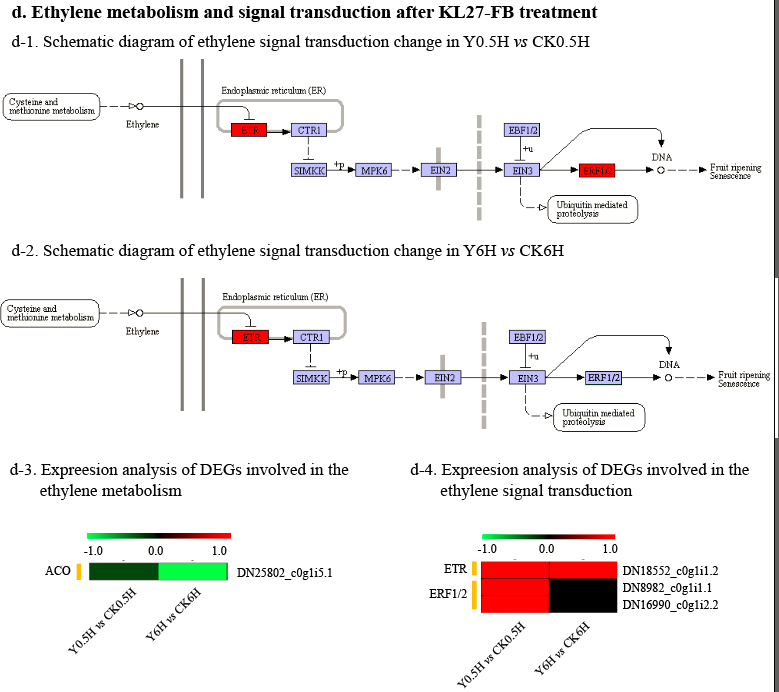

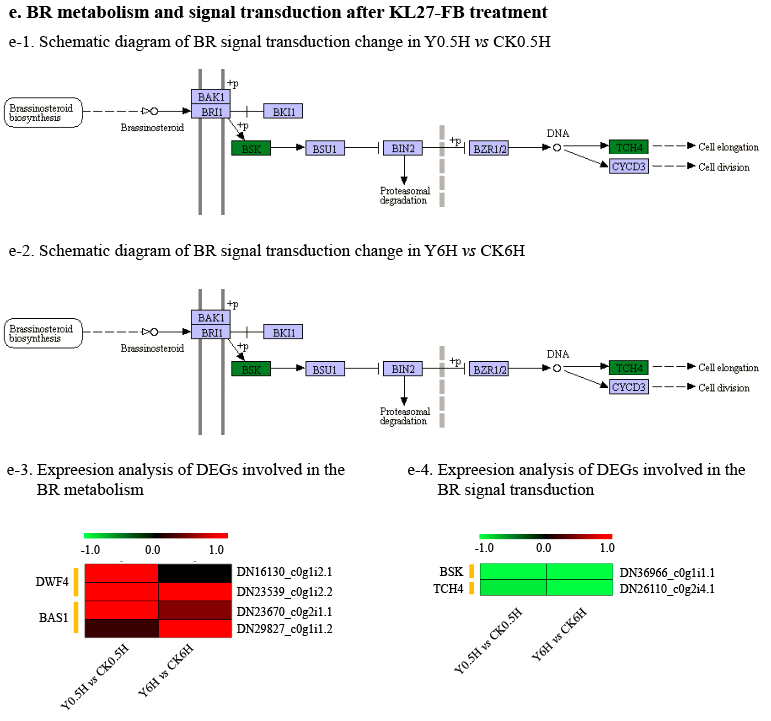

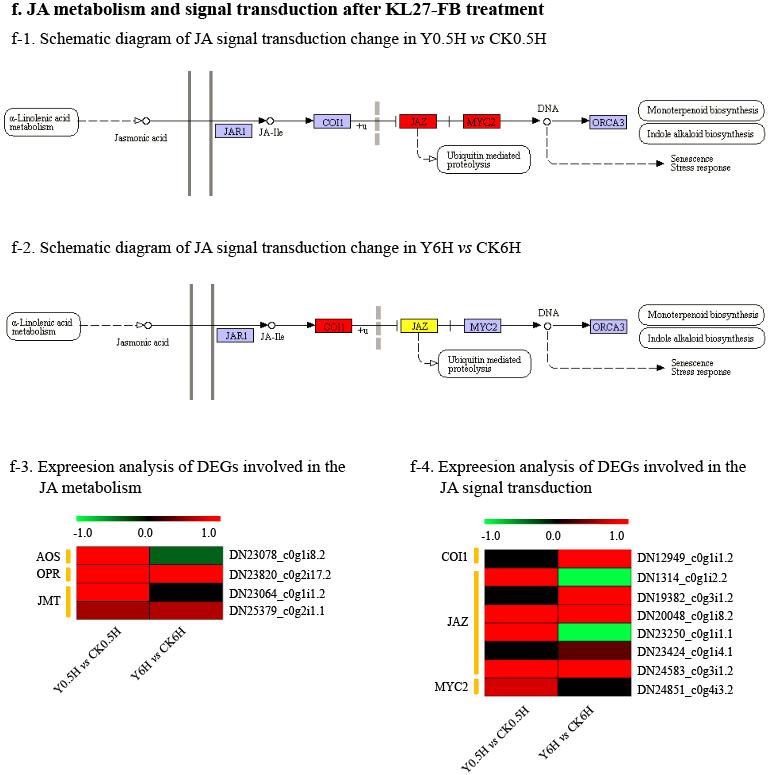

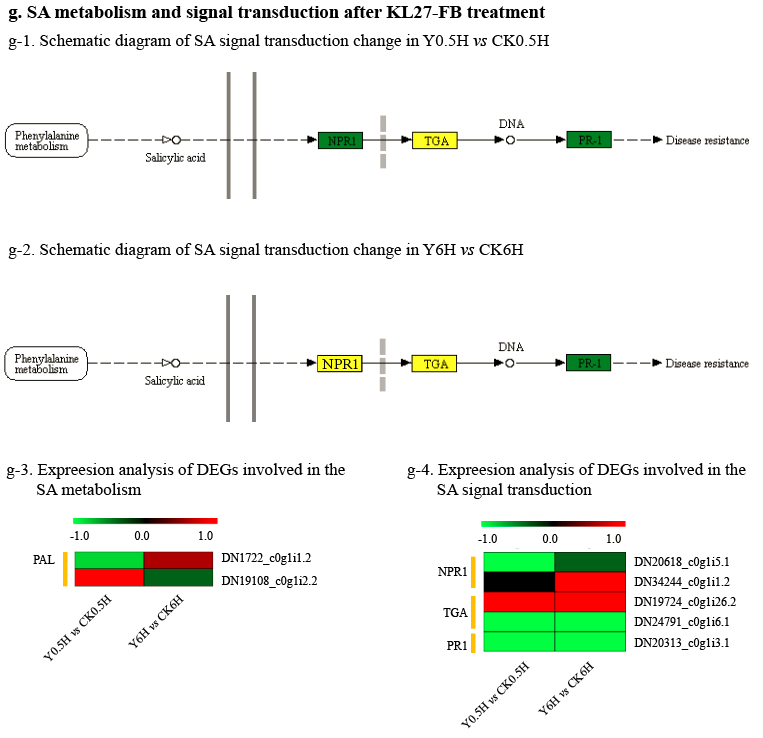

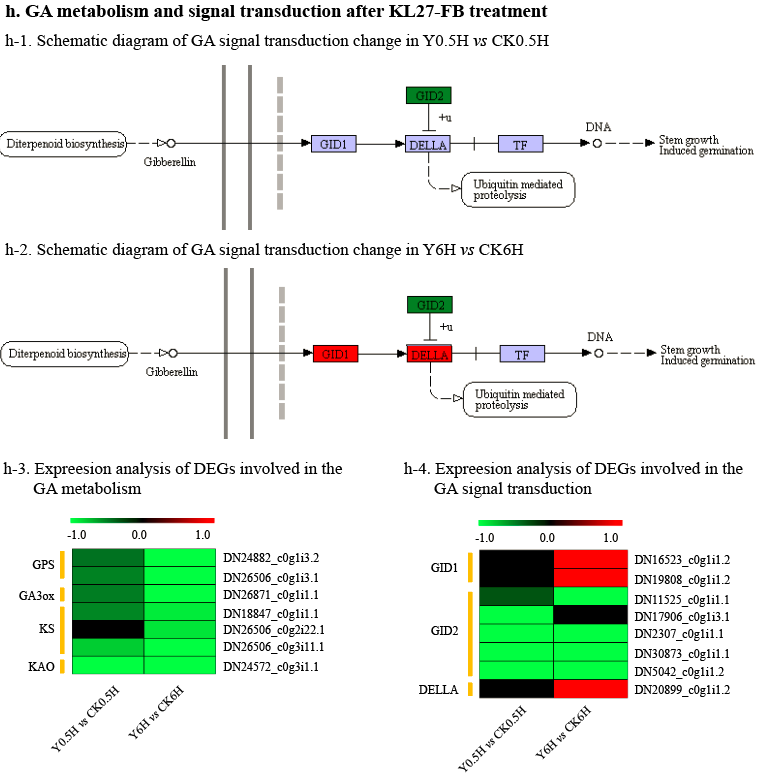


Figure S4 Hormone metabolism and signal transduction of auxin (a), CTY (b), ABA (c), ET (d), BR (e) , JA (f) , SA (g) and GA (h) after KL27-FB treatment. The expression fold change of each genes in the two comparisons were shown by a heatmap. The bar indicated the the “log2(fold change)”.
