# Supplementary material for: Endophytic fungus Pseudodidymocyrtis lobariellae KL27 promotes taxol biosynthesis and accumulation in Taxus chinensis
Source: BMC Plant Biol. 2022 Jan 3;22:12. doi: 10.1186/s12870-021-03396-6 (PMC8722197; doi:10.1186/s12870-021-03396-6)
Supplement: Supplementary file 14 — Additional file 14: Figure S5. The expression change of each TF-encoding genes in the two comparisons was shown by a heatmap. [file 12870_2021_3396_MOESM14_ESM.doc]

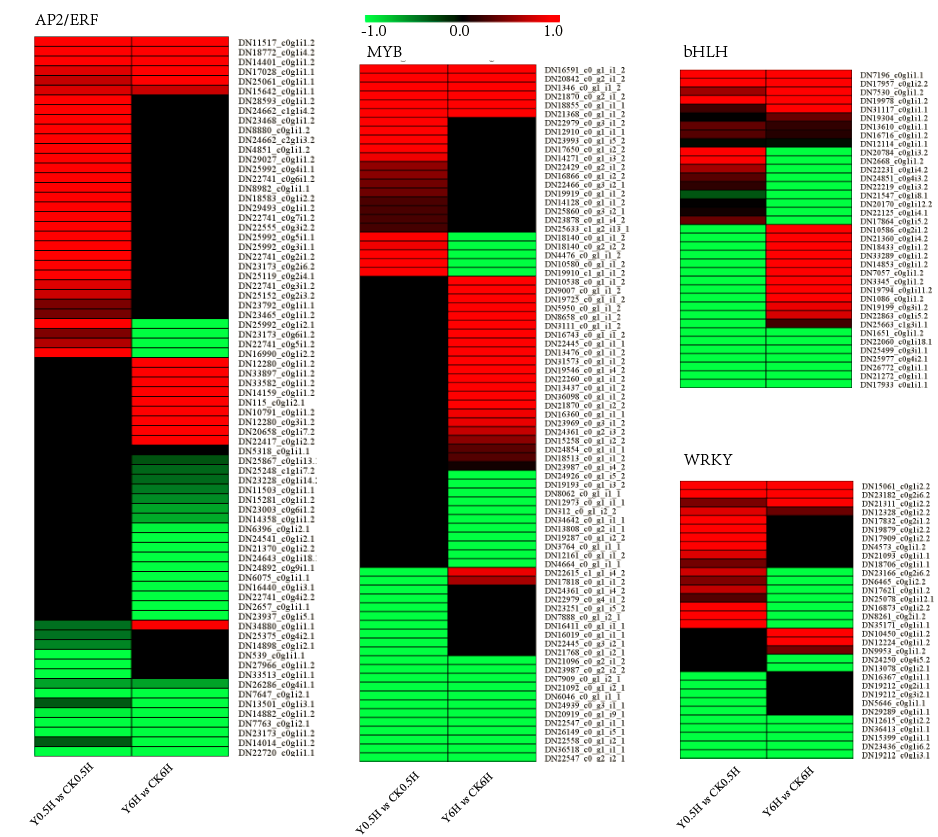


Figure S5 The expression change of each TF-encoding genes in the two comparisons was shown by a heatmap. The bar indicated the the “log2(fold change)”.
