# Supplementary material for: Endophytic fungus Pseudodidymocyrtis lobariellae KL27 promotes taxol biosynthesis and accumulation in Taxus chinensis
Source: BMC Plant Biol. 2022 Jan 3;22:12. doi: 10.1186/s12870-021-03396-6 (PMC8722197; doi:10.1186/s12870-021-03396-6)
Supplement: Supplementary file 15 — Additional file 15: Table S8. MYC2, MYC3 and MYC4 regulate effect on the taxol biosynthesis genes. [file 12870_2021_3396_MOESM15_ESM.doc]

Table S8 MYC2, MYC3 and MYC4 regulate effect on the taxol biosynthesis genes.

| Gene | MYC2 | MYC3 | MYC4 |
| --- | --- | --- | --- |
| TBT | + | + | + |
| TAT | / | / | / |
| DBTNBT | + | + | ++ |
| T2OH | +++ | ++ | ++ |
| T7OH | +++++ | +++ | +++++ |
| T10OH | + | + | ++ |
| PAM | + | + | + |
| TS | ++ | + | + |
| T5OH | + | + | + |
| T13OH | ++ | ++ | + |
| DBAT | + | + | + |
| BAPT | + | - | / |
| GGPPS | + | / | + |
| T14OH | / | - | / |

“/”represents no regulate effect, “+” represents have up-regulated effect, “-” represents have down-regulate effect, the number of “+” and “-” represents the strength.

This table was drown according to the literature [1].

**Reference**

1. Cui Y, Mao R, Chen J, Guo, Z. Regulation mechanism of myc family transcription factors in jasmonic acid signalling pathway on taxol biosynthesis. Int J Mol Sci. 2019;20(8).
